# Waste Cooking Oil as Eco-Friendly Rejuvenator for Reclaimed Asphalt Pavement

**DOI:** 10.3390/ma17071477

**Published:** 2024-03-24

**Authors:** Noemi Bardella, Manuela Facchin, Eleonora Fabris, Matteo Baldan, Valentina Beghetto

**Affiliations:** 1 Crossing S.r.l., Viale della Repubblica 193/b, 31100 Treviso, Italy; noemi.bardella@crossing-srl.com; 2 Department of Molecular Sciences and Nanosystems, University Ca’ Foscari of Venice, Via Torino 155, 30172 Venice, Italymatteo.baldan@unive.it (M.B.); 3 Consorzio Interuniversitario per le Reattività Chimiche e la Catalisi (CIRCC), Via C. Ulpiani 27, 70126 Bari, Italy

**Keywords:** waste cooking oil, reclaimed asphalt pavement, waste valorisation, circular economy

## Abstract

Over 50 MioT of Waste Cooking Oil (WCO) was collected worldwide in 2020 from domestic and industrial activities, constituting a potential hazard for both water and land environments, and requiring appropriate disposal management strategies. In line with the principles of circular economy and eco-design, in this paper an innovative methodology for the valorisation of WCO as a rejuvenating agent for bitumen 50/70 coming from Reclaimed Asphalt Pavement (RAP) is reported. In particular, WCO or hydrolysed WCO (HWCO) was modified by transesterification or amidation reactions to achieve various WCO esters and amides. All samples were characterised by nuclear magnetic resonance, melting, and boiling point. Since rejuvenating agents for RAP Cold Mix Asphalt require a melting point ≤0 °C, only WCO esters could further be tested. Efficiency of WCO esters was assessed by means of the Asphaltenes Dispersant Test and the Heithaus Parameter. In particular, bitumen blends containing 25 wt% of WCO modified with 2-phenylethyl alcohol, showed high dispersing capacity in *n*-heptane even after a week, compared to bitumen alone (1 h). Additionally, the Heithaus Parameter of this bitumen blend was almost three times higher than bitumen alone, further demonstrating beneficial effects deriving from the use of WCO esters as rejuvenating agents.

## 1. Introduction

Waste Cooking Oil (WCO) is a domestic and industrial waste obtained from food cooking [1]. The term WCO generically refers to frying oil used at high temperatures and edible fats mixed with food waste and water, which can no longer be used for cooking [2]. WCO is prevalently obtained from household, commercial, and industrial by-products, and derives mainly from palm, soybean, canola, sunflower, peanut, cottonseed, coconut, olive, and corn oil [3].

Without proper disposal, WCO is highly polluting for both water and land environments, requiring appropriate disposal management [4]. It is important to note that WCO annual production and recovery methods vary consistently, making estimations of WCO yearly collection difficult. For example, according to European Union (EU) estimations, around 8 L WCO/capita/year should be collected, which, considering a total EU population of around 500 million, corresponds to 4 Miot/year of WCO. As a matter of fact, these data are seven times higher than the current collected amount in the EU, clearly evidencing the gap between produced and collected WCO [5]. This considered, in 2020, the amount of WCO collected worldwide was about 50 Miot [2,6,7,8], of which Europe was responsible for approximately 1 Miot [9]. The United States is the world’s leading producer of WCO [10] with 10 Miot/year collected, followed by China, where between 40 and 60% WCO is not disposed of or is illegally reused in kitchens [2]. Despite this, the Chinese government is making efforts to promote the correct disposal and reuse of WCO to produce biodiesel and has promulgated several regulations, including Regulations on Waste Cooking Oil Management, promoting WCO recovery and recycling [2].

As far as the EU is concerned, starting from the 1970s, with the waste oil directive 75/439/EEC, the European Commission committed to collecting used oil in order to limit environmental damage and promote recovery and recycling technologies and any derived markets. Recently, all EU member states have undersigned the Renewable Energy Directive, in which they committed to enhance the use of WCO for biofuels from 3.5% to 5.5% by 2023 [11].

Italy produces approximately 260.000 t/year of WCO at the industrial level (restaurants, catering), which are collected by CONOE (National Consortium for the collection and treatment of used oil), and used for the production of soaps, additives for lubricating oils, and biodiesel [12]. On the contrary, the proper recovery and disposal of WCO produced at the domestic level is on a voluntary basis; therefore, WCO may be released into the sewer system.

Adequate collection of WCO is not only beneficial for the environment but is also economically advantageous since WCO can be used to obtain biofuels and other high-value chemicals [13,14]. The need to recover and recycle by-products or end-of-life products, reducing primary resource consumption, is becoming increasingly urgent, leading to a change from a linear economy based on a “take-make-discard” model to a circular economy based on green chemistry and eco-design [15,16,17,18,19,20].

Although WCO is mainly employed at the industrial level for the production of biofuels, due to its similar elemental composition to petroleum, it has also received considerable attention for its potential use as fluxing or rejuvenating agent for aged bitumen [21,22]. According to Uz and Gökalp, the use of WCO rejuvenating agents has beneficial effects on aged bitumen recovered from Reclaimed Asphalt Pavement (RAP) that would otherwise be discarded [21].

The presence of variable quantities of free fatty acids (FFAs) is responsible for increasing acidity content in WCO which influences its performances. Specifically, when WCO is employed for the production of bitumen blends, acidity content negatively influences the interaction between WCO and bitumen [20,21]. Reducing the FFAs in WCO by pretreatment techniques such as esterification or refining processes can promote the overall performance of WCO-modified bitumen. Although the use of WCO as additive for bitumen modification has been previously reported in the literature [23,24], only a few studies report the possibility to achieve higher performances, using chemically modified WCO by transesterification [25,26]. In particular, Oldham and coworkers recently reported that a reduction in WCO acidity content by transesterification with methanol significantly improved its efficiency for the production of bitumen blends employed as rejuvenators for RAP, improving asphalt recycling [26]. Similar results were also reported by Wan Azahar and coworkers with WCO prepared by alkali-promoted esterification in the presence of methanol [27].

**Figure 1:**
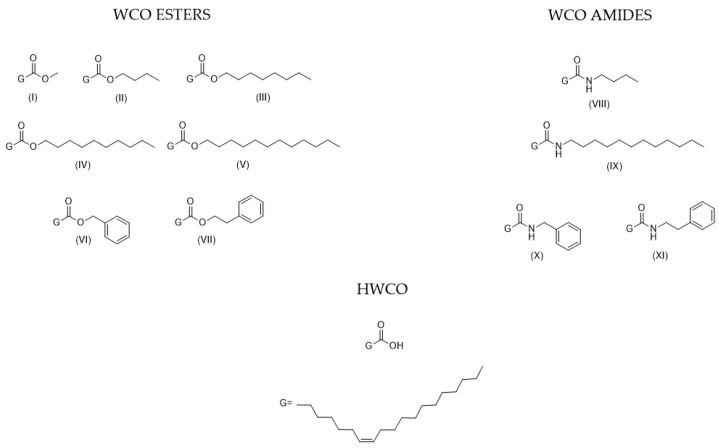
Esters and amides obtained from chemical modification of WCO and HWCO (Hydrolysed WCO).

Within this panorama, the aim of this work is to study a wide range of chemical modifications of WCO by transesterification or amidation reactions with aliphatic and aromatic alcohols or amines, having alkyl chains of variable length (Figure 1) [28].

All products prepared were characterised by ^1^H and ^13^C Nuclear Magnetic Resonance (NMR) and their determined melting and boiling points. Preliminary data to determine the efficacy of WCO modified by transesterification and amidation as rejuvenating agents for aged bitumen were found by using the Asphaltene Dispersant Test and the measurement of Heithaus Parameter.

## 2. Materials and Methods

### 2.1. General Remarks

**Figure 2:**
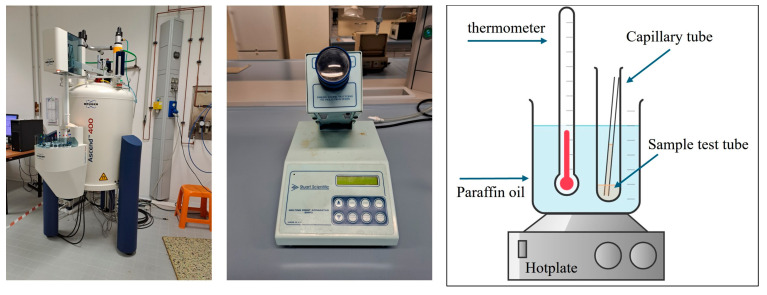
Pictures of a Bruker Ultra Shield 400 NMR spectrometer (**left**), a Stuart Scientific SMP3 melting point apparatus (**center**) and apparatus set up for boiling point determination (**right**).

All commercially available reagents, solvents and chemicals were provided by Sigma Aldrich (Milan, Italy) and used as received. WCO was delivered by Elite Ambiente (Grisignano di Zocco, VI, Italy). Aged bitumen was extracted from RAP, a kind gift from IFAF Spa (Noventa di Piave, Italy). ^1^H and ^13^C NMR spectra of the products were registered on a Bruker Ultra Shield 400 spectrometer operating at 400.0 and 101.0 MHz, respectively, and are reported in the Supplementary Materials. Nuclear Magnetic Resonance (NMR) spectroscopy is an analytical technique used to determine the content and purity of a sample as well as its molecular structure. A picture of a Bruker Ultra Shield 400 NMR spectrometer is reported in Figure 2 (left) Melting points were measured with a Stuart Scientific SMP3 (Figure 2 center). Samples were loaded into a sealed capillary tube, which was then placed in the apparatus. The sample was then heated with an oil bath and, as temperature increases, the sample is observed to determine when the phase change from solid to liquid occurs.

Boiling points were measured according to the Capillary Method [29]. Samples were placed in a capillary, sealed at one end, and opened at the other. The capillary was placed in a liquid with the open side facing downwards and heated. As the temperature increases, the air in the capillary escapes and is replaced by the vapor of the liquid and vapor pressure in the capillary increases. Once it exceeds the atmospheric pressure, the vapor escapes the capillary in a stream of bubbles. When the heat is removed, the liquid cools, and the vapor pressure in the capillary decreases. When the vapor pressure reaches the atmospheric pressure, the liquid begins to fill the capillary. The temperature at which this occurs is the boiling point. The apparatus set up for boiling point by capillary method is reported in Figure 2 right.

### 2.2. Cooking Oil Purification and Chemical Modification

Prior to chemical reactions WCO was purified as follows: to a 1 L bottom round flask, equipped with a magnetic stirrer, 500 g of WCO, 30 g of activated charcoal DARCO^®^, 100 mesh particle size, powder (6 wt%), and 130 mL dichloromethane (CH_2_Cl_2_) (35 wt%) were added and stirred for 3 h at room temperature. Then, WCO was filtered on paper to remove solid cooking residues and activated charcoal, and dichloromethane was evaporated [30]. Purified WCO, recovered in the form of an amber-coloured liquid, was characterized by ^1^H NMR, and ^13^C NMR (see Supplementary Materials).

#### 2.2.1. Transesterification of WCO

**Scheme 1:**
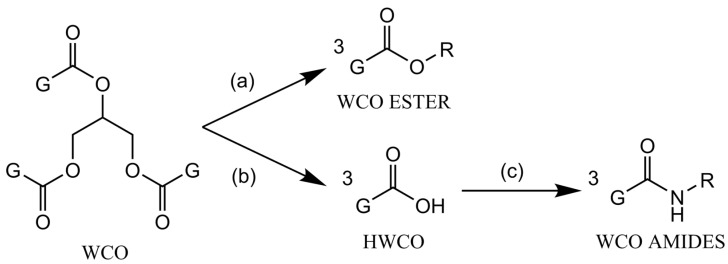
Synthetic strategies for the preparation of WCO esters and amides. (**a**) ROH, NaOMe, neat, reflux; (**b**) NaOH 4.5 M, 60 °C, 8 h/HCl 5 M; (**c**) RNH_2_ in toluene, 110 °C, 48 h, Dean–Stark apparatus.

Seven different esters were prepared by transesterification of WCO with seven different alcohols, specifically methyl, *n*-butyl, octyl, decanoyl, dodecyl, benzyl, and phenylethyl alcohol (Scheme 1). The general synthesis procedure is as follows: to a 250 mL round bottom flask, equipped with a magnetic stirrer, 5 g of purified WCO and 20 wt% of the selected alcohol were added and stirred for 5 min at room temperature. Then, 0.87 g (16.2 mmol) of sodium methoxide was added and the temperature was raised to that of the alcohol boiling point for 2 h for (**I**), 4 h for (**II**), 48 h for (**III**), and (**IV**) and 24 h for (**V**), (**VI**), and (**VII**) [31]. The crude products were poured in dichloromethane and washed with brine, then the organic phase was dried over anhydrous magnesium sulphate. After filtration and solvent evaporation under reduced pressure, the products were obtained in yields between 60 and 98%, depending on the alcohol employed (see Table 1). Purified products were characterised by ^1^H NMR and ^13^C NMR in CDCl_3_ (see Supplementary Materials). Melting and boiling points were measured and are reported in Table 1.

#### 2.2.2. Purified WCO Hydrolysis

For amidation reactions, WCO was first hydrolysed to obtain the free fatty acid structure (HWCO) and then amidated with different types of amines: *n*-butyl, dodecyl, benzyl, and phenylethyl amine (Scheme 1). The general synthesis procedure is as follows: to a 100 mL round bottom flask, equipped with a magnetic stirrer, 20 g of purified WCO and 3.6 g of a 4.5 molar concentration water solution of sodium hydroxide (NaOH 4.5 M) were added and stirred for 8 h at 60 °C. Then, hydrochloric acid (HCl) 5 M was added until pH 2–3 was reached. Then, the aqueous mixture was washed with diethyl ether, the organic fraction collected and dried over anhydrous magnesium sulphate. After filtration and solvent removal under reduced pressure, a white solid product was obtained in a 67% yield.

#### 2.2.3. Amidation Reactions

**Figure 3:**
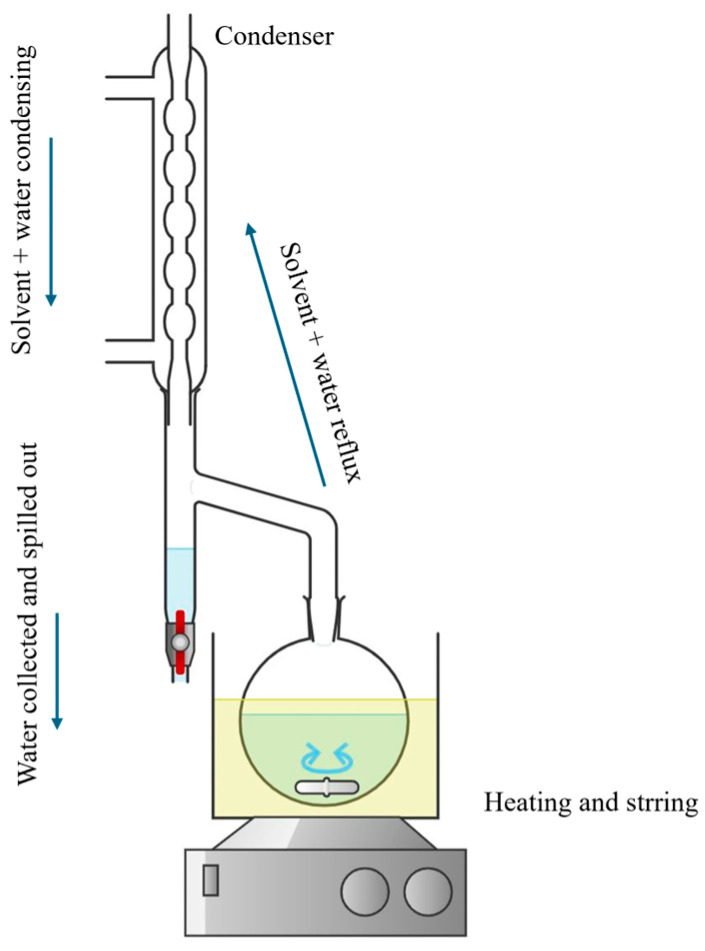
Dean–Stark set up for the transesterification of purified WCO.

Amidation reactions were carried out employing a Dean–Stark Apparatus (Figure 3). A Dean–Stark apparatus is used in combination with a reflux condenser and a distillation flask for the separation of water from other solvents presents (for example toluene). A Dean–Stark apparatus allows continuous removal of the water produced during esterification reactions carried out at reflux temperature, promoting the formation of products [32].

The procedure employed was the same both for aliphatic and aromatic amines (Scheme 1). To a 50 mL round bottom flask, equipped with a magnetic stirrer and Dean–Stark apparatus, 2.5 g of hydrolysed WCO was dissolved in 50 mL of toluene. Then, 20 wt% of the selected amine were added and stirred at reflux for 48 h. Toluene was evaporated and the crude solid product poured into dichloromethane, washed three times with NaOH 1 M, and two times with distilled water. The organic fraction was then dried over anhydrous magnesium sulphate. After filtration and solvent removal under reduced pressure, a solid product was obtained in yields between 45 and 92% depending on the type of amine employed (see Table 1). Purified products were characterised by ^1^H NMR and ^13^C NMR in CDCl_3_ (see Supplementary Materials). Melting and boiling points were measured and are reported in Table 1.

#### 2.2.4. Asphaltene Dispersant Test (ADT)

WCO esters were added with a weight ratio of 25 wt% by the weight of aged bitumen 50/70 [33]. RAP employed in this work was derived from asphalt prepared with bitumen 50/70, paved, and reclaimed by IFAF S.p.a. Aged bitumen was recovered from RAP by Soxhlet extraction at 40 °C for 4 h with dichloromethane. According to the literature [34,35], all aged bitumen blends were stirred at 500 rpm with a magnetic stirrer to homogenise the components; consequently, 0.6 mL of the mixture was dissolved in 19.4 mL of *n*-heptane. A sample of crude bitumen was used as reference. All specimens were introduced into graduated centrifuge tubes and left standing for a specific period of time, also called aging time (1 h, 24 h and 1 week). The rejuvenating effect of WCO esters was determined by comparing the translucency of the glass and the amount of sediment at different aging times with respect to the reference bitumen.

#### 2.2.5. Heithaus Parameter Measurement

This method was developed by Heithaus [36,37] to predict the stability of aged bitumen and heavy crude oils. A selected amount of bitumen is dissolved in different volumes of toluene and *n*-heptane is added until flocculation occurs. Flocculation point is monitored by deposing few drops of the titrated solution on filter paper; if a dark precipitate inside the drop on the filter paper is observed, the flocculation point is achieved. Once the volume of *n*-heptane needed for flocculation is known (V_t_, mL), the concentration of bitumen in the total volume (C, g/mL) and flocculation ratio (FR) can be calculated according to Equation (1):

(1) $$C=\frac{W_{A}}{V_{s}+V_{t}}\;,\;FR=\frac{V_{s}}{V_{s}+V_{t}}\;$$

where W_A_ (g) corresponds to the mass of the aged bitumen weighted, and V_S_ (mL) corresponds to the volume of toluene introduced into the solution. C and FR data are plotted, and C_min_ and FR_max_ are determined (see Supplementary Materials). C_min_ is the onset of asphaltene precipitation (OAP, mL/g) corresponding to the minimum volume of *n*-heptane causing bitumen precipitation in the absence of toluene [36,38]. FR_max_ corresponds to the ratio of solvent to titrant at which bitumen is soluble in all concentrations. Thus, P_α_, P_0_, and P are measured, according to Equations (2)–(4):

(2) $$P_{a}=1-{FR}_{max},$$

(3) $$P_{0}={FR}_{max}\cdot \left[\frac{1}{C_{min}}+1\right],$$

(4) $$P=\frac{P_{0}}{1-P_{a}}$$

P_α_ and P_0_ correspond to asphaltenes and maltenes solubility, respectively, and allow us to calculate P, the Heithaus Parameter. If P > 1, the bitumen is stable and does not flocculate, while if P < 1, bitumen is not stable and flocculates. For the measurement, 1 g of aged bitumen (with or without 25 wt% of WCO ester) was dissolved in 1.00 mL, 1.25 mL, 1.50 mL, and 2.00 mL of toluene and titrated with *n*-heptane until the flocculation point of the asphaltenes was reached. Tests were performed in triplicate. Calculations are reported in the Supplementary Materials.

## 3. Results and Discussion

**Figure 4:**
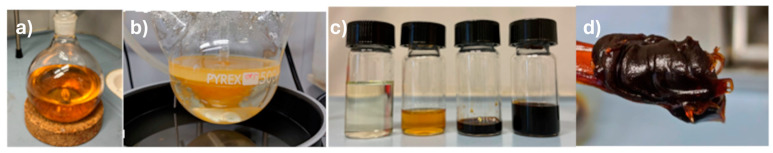
Pictures of purified WCO (**a**), WCOH (**b**), methyl, butyl, octyl, and dodecyl WCO esters (**c**) and butyl amide (**d**).

Oil mixtures received from Elite Ambiente derive from industrial or household operations and contain different commercially available vegetable oils. Due to the variability in WCO, no analytical characterization of the mixture was carried out to determine the composition of the specific batch under analysis, and all the data reported in this paper refer to tests carried out with the same sample of WCO [39,40,41,42,43,44,45]. Pictures of WCO, HWCO, and chemically modified WCO are reported in Figure 4.

### 3.1. WCO Chemical Modification

Purified WCO was chemically modified according to the two different strategies reported in Scheme 1. The first one foresees direct transesterification of purified WCO to obtain different esters, the latter instead requires two steps, i.e., hydrolysis of WCO (HWCO) carried out with a Dean–Stark apparatus (see Section 2) followed by amidation to obtain different amides.

WCO hydrolysis was carried out in the presence of an aqueous solution of NaOH 4.5 M at 60 °C [46], and after work up HWCO was recovered with 70% yield.

Esters were prepared by the transesterification reaction, mixing WCO with an alcohol at reflux temperature for several hours (see Table 1) [46]. For WCO esters, the highest yields were obtained from the reaction of WCO with methanol due to its reduced steric hindrance and high nucleophilicity, while decanol, benzyl alcohol, and phenylethyl alcohol were partially recovered unreacted even if reaction times were prolonged up to 24 h (see Table 1).

Amides, instead, were prepared by reacting HWCO with an amine in toluene at reflux, for 48 h. In agreement with the literature highest yields in WCO amides were achieved in the presence of benzylamine or phenethylamine (see Table 1), because of their higher nucleophilicity compared to other amines tested [47,48]. As mentioned above, in this work for all WCO additives, melting and boiling points were measured and are reported in Table 1. From these data, it emerges that all additives have similar boiling points (>200 °C), while melting points differ significantly between esters and amides. In fact, WCO esters all have melting points around 0 °C, while WCO amides have higher m.p., ranging between 48 °C and 92 °C (see Table 1).

These higher temperatures are probably to be ascribed to the hydrogen bonds that are established between the protons and the nitrogen electron pairs in the amides [34]. Since it is widely known from the literature that solid additives cannot be used for Cold Mix Asphalt preparation, WCO amides were not further investigated for this application. Amides could be suitable for Warm Mix Asphalt and Hot Mix Asphalt, and thus future studies will focus on this application.

On the contrary, thanks to their low melting point, WCO esters appeared to be interesting candidates as additives for Cold Mix Asphalt preparation, and so tests for this specific application were performed.

Based on their different applications, asphalts can be classified into Hot Mix Asphalt (HMA), Warm Mix Asphalt (WMA), and Cold Mix Asphalt (CMA) [49,50]. Considering costs and environmental sustainability [51], CMA is considered to be highly efficient since it requires lower processing temperatures (between 0 °C and 40 °C) and easily available equipment. Since prevention, recycling and reuse is highly encouraged to face the existing environmental issues of waste generation [52], CMA appears to be particularly interesting since it allows recycling of reclaimed asphalt pavement (RAP). RAP is obtained from the removal of aged road pavement and thus its reuse generates both environmental and economic advantages [53,54]. Babashamsi et al. highlighted that by incorporating between 30 wt% and 50 wt% RAP into CMA, savings of up to USD 58,000/km may be achieved [55]. Asphalt mixtures consist of two valuable non-renewable resources [56], both subject to aging processes: aggregates and bitumen. Aggregates are inert rock materials which reduce the amount of bitumen required in the asphalt preparation and can have different grain sizes such as sand and crushed stone [57]. Bitumen is a viscous fluid in which aggregates of polar macromolecules (asphaltenes) are dispersed in a more apolar continuous phase (maltenes). This fraction is composed of aromatics, resins, and saturates which, during asphalt processing, undergo different chemical modifications [58,59,60]. In fact, when bitumen is mixed with aggregates [49,61,62] to produce asphalt for pavement constructions, it starts to age and loses its original physical mechanical characteristics. Polymerization and oxidation [63] reactions are among the main causes for maltenes reduction, which results in aggregation into larger macromolecules [58,64]. This process stiffens the material, making it more brittle [58].

Rejuvenating additives may help to solve this problem, promoting recycling and reuse of aged bitumen contained into RAP. These additives are mostly amphiphilic molecules composed of a non-polar tail and a highly aromatic polar head [24]. Their amphiphilic nature contributes to reduce bitumen aging since their polar head interacts with asphaltene aggregates de-agglomerating them, while their non-polar tail enriches the maltenes fraction [34,61], replenishing the volatiles and renewing the chemical–physical characteristics of bitumen [24,65].

Within this panorama, all WCO ester additives synthetised in this paper are potentially rejuvenating agents for aged bitumen, possessing both polar head and a non-polar tail (WCO) together with a low melting point. Preliminary evaluations on the use of WCO esters was carried out with additives (**II**), (**VI**) and (**VII**) to verify their potential rejuvenating properties by the Asphaltenes Dispersant Test [34] and the measurement of the Heithaus Parameter [36].

### 3.2. Asphaltene Dispersant Test (ADT)

**Figure 5:**
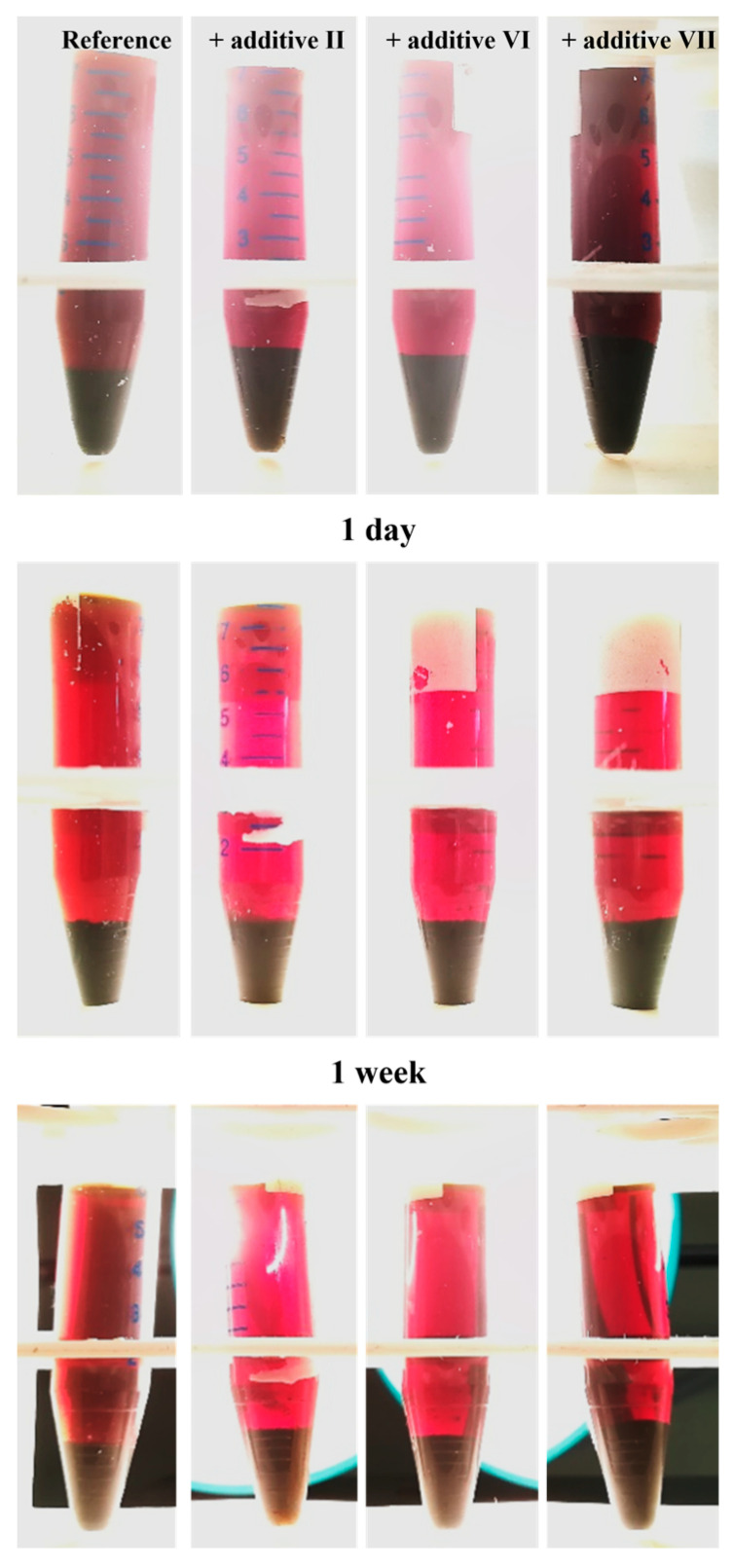
Deposition of asphaltenes observed after 1 h, 1 day, and 1 week in *n*-heptane; from left to right: aged bitumen (reference), aged bitumen enriched with 25 wt% of additive (**II**), aged bitumen enriched with 25 wt% of additive (**VI**), and aged bitumen enriched with 25 wt% of additive (**VII**).

To evaluate the rejuvenating efficacy of additives (**II**), (**VI**), and (**VII**), the precipitation of the asphaltenes fraction present in aged bitumen was observed after one hour, one day, and one week (see Figure 5), and compared to the reference. According to the Asphaltene dispersant test, a rejuvenating agent reduces flocculation phenomena and improves the dispersion of aged bitumen in *n*-heptane [34]. As shown in Figure 5, after just 1 h, asphaltenes present in aged bitumen alone begin to precipitate, occupying a volume of 0.5 mL into the reference tube. On the contrary, after 1 h, bitumen samples containing 25 wt% of ester (**II**), (**VI**) and (**VII**) occupy a higher volume (about 1 mL), indicating an improved dispersion efficiency. After one day, no variation was observed in tubes containing the reference sample and aged bitumen blend with additive (**VII**), while asphaltenes occupied a lower volume in tubes containing additives (**II**) and (**VI**) (approximately 0.7 mL).

After one week, no significant variations were observed in the reference tube, while the volume occupied by the precipitated asphaltenes was further reduced to 0.5 mL in tubes containing additives (**II**) and (**VI**). Interestingly, in samples containing additive (**VII**), asphaltenes occupied a volume of 0.7 mL, suggesting an improved dispersing capacity of this additive even after a week. It may be supposed that due to its aromatic nature, additive (**VII**) reintegrates the maltene fraction in the aged bitumen, with a potential rejuvenating effect. Additionally, properties shown by (**VII**) could be ascribed to the fact that, compared to (**VI**), the ester moiety is further away from the aromatic ring and, therefore, less encumbered [34]. Further tests were thus carried out only on best performing additive (**VII**).

### 3.3. Calculation of the Heithaus Parameter

The Heithaus parameter (P) provides information on the stability of aged bitumen and of the aged bitumen blends enriched with an additive, giving indications on whether the mixture is in equilibrium or tends to flocculate. To measure P, two different sets of experiments were carried out and compared: the first one was performed on a sample of crude aged bitumen, and the second one on a sample of aged bitumen enriched with 25 wt% of additive (**VII**) by weight of bitumen. The Heithaus Parameter (P) is measured by Equations (1) and (2), together with linear correlation, as described in the experimental section. If P > 1, the bitumen is stable and does not flocculate, while if P < 1, bitumen is not stable and flocculates. From these experiments, it was possible to determine that the Heithaus Parameter for aged bitumen is of 1.48, while when additive (**VII**) was present, the Heithaus parameter increased to 2.73 (see Supplementary Materials). This value is almost double compared to that of the aged bitumen alone, indicating that additive (**VII**) is a rejuvenating agent, improving aged bitumen stability.

## 4. Conclusions

In conclusion, in this paper, an innovative methodology for the modification of WCO was reported together with its use as rejuvenating agent for asphalt pavement. In particular, a set of different alcohols and amines were used to modify WCO or hydrolysed WCO (HWCO) by transesterification or amidation reaction. All WCO and HWCO derivatives were characterised by ^1^H and ^13^C NMR. Melting and boiling temperatures of WCO derivatives were used to select potentials of rejuvenating agents to be used for RAP recycling for Cold Mix Asphalt production. Since only compounds with m.p. ≤ 0 °C may be used for the scope, only WCO esters (**II**, **VI,** and **VII**) were further tested by the Asphaltenes Dispersant Test [34] and the Heithaus Parameter [35], to verify their efficiency as rejuvenating agents. From these experiments, it emerged that additive (**VII**) highly improved the dispersing capacity of bitumen in *n*-heptane even after a week, compared to the reference sample (1 h). Additionally, the Heithaus Parameter of bitumen blends containing 25 wt% of WCO modified with 2-phenylethyl alcohol (**VII**) was almost three times higher than bitumen alone, further confirming that (**VII**) has good potential efficiency as a rejuvenating agent. These results are very encouraging and indicate that WCO may be transformed into a valuable starting material for the production of bio-based rejuvenating agents for asphalt pavement production. Further studies will be carried out on asphalt pavement prepared with aged bitumen blends reported in this work in order to assess the influence on surface texture and noise emissions [66]. Additionally, further tests are ongoing to achieve information on the efficiency of WCO amides for Hot Mix Asphalt modification.

## Figures and Tables

**Table 1:** Reaction conditions and yields for the synthesis of WCO esters and amides and boiling/melting points.

	Product	Optimum Reaction Condition	Yield (%)	b.p. (°C)	m.p. (°C)
Esters ^a^					
CH_3_OH	**I**	65 °C, 2 h	72	>200	0.6
*n*-C_4_H_5_OH	**II**	118 °C, 4 h	50	>200	0.6
C_8_H_17_OH	**III**	188 °C, 48 h	58	>200	0.4
C_10_H_21_OH	**IV**	235 °C, 24 h	34	>200	0.4
C_12_H_25_OH	**V**	250 °C, 24 h	34	>200	0.3
PhCH_2_OH	**VI**	205 °C, 24 h	36	>200	0.2
PhCH_2_CH_2_OH	**VII**	225 °C, 24 h	34	>200	0.2
Amides ^b^					
*n*-C_4_H_5_NH_2_	**VIII**	110 °C, 48 h	69	>200	48
C_12_H_25_NH_2_	**IX**		44	>200	73
PhCH_2_NH_2_	**X**		92	>200	90
PhCH_2_CH_2_NH_2_	**XI**		54	>200	92

^a^ Reaction Conditions: Solvent: neat; alcohol to WCO weight ratio: 1/5; Catalyst: NaOMe. ^b^ Reaction Conditions: Solvent: Toluene; toluene to HWCO weight ratio 7/1; amine to HWCO weight ratio: 1/5.

## Data Availability

Data are contained within the article and Supplementary Materials.

## Supplementary Materials

The following Supplementary Materials can be downloaded at: https://www.mdpi.com/article/10.3390/ma17071477/s1, Figures S1–S28: ^1^H and ^13^C NMR spectra of additives; Table S1: Mass of bitumen, V_S_, concentration, V_T_, C and FR for three samples containing different concentrations of bitumen without additives.; Table S2: Mass of bitumen, V_S_, concentration, V_T_, C and FR for the four samples with different concentrations of bitumen containing 25 wt% of additive (**VII**).

## References

[1] Iglesias L., Laca A., Herrero M., Díaz M. (2012). A life cycle assessment comparison between centralized and decentralized biodiesel production from raw sunflower oil and waste cooking oils. J. Clean. Prod..

[2] Zhang H., Wang Q., Mortimer S.R. (2012). Waste cooking oil as an energy resource: Review of Chinese policies. Renew. Sustain. Energ. Rev..

[3] Shahbandeh M. Vegetable Oils and Fats—Statistics & Facts, Statista 2024. https://www.statista.com/topics/2025/us-vegetable-oils-and-fats/#editorsPicks.

[4] Frota De Albuquerque Landi F., Fabiani C., Castellani B., Cotana F., Pisello A.L. (2022). Environmental assessment of four waste cooking oil valorisation pathways. Waste Manag..

[5] Used Cooking Oil. https://www.eubia.org/cms/wiki-biomass/biomass-resources/challenges-related-to-biomass/used-cooking-oil-recycling/.

[6] Awogbemi O., Idoko E.O., Inambao F.L. (2019). Comparative study of properties and fatty acid composition of some neat vegetable oils and waste cooking oils. Int. J. Low-Carbon Technol..

[7] Awogbemi O., Von Kallon D.V., Aigbodion V.S. (2021). Advances in biotechnological applications of waste cooking oil. Case Stud. Chem. Environ. Eng..

[8] Foo W.H., Koay S.S.N., Chia S.R., Chia W.Y., Tang D.Y.Y., Nomanbhay S., Chew K.W. (2022). Recent advances in the conversion of waste cooking oil into value added products: A review. Fuel.

[9] Mannu A., Ferro M., Di Pietro M.E., Mele A. (2019). Innovative applications of waste cooking oil as raw material. Sci. Prog..

[10] Goh B.H.H., Chong C.T., Ge Y., Ong H.C., Ng H., Tian B., Ashokkumar V., Lim S., Seljak T., Józsa V. (2020). Progress in utilization of waste cooking oil for sustainable biodiesel and biojet fuel production. Energy Convers. Manag..

[11] Special Report 29/2023: The EU’s Support for Sustainable Biofuels in Transport—An Unclear Route ahead. http://data.europa.eu/eli/C/2023/1598/oj.

[12] CONOE. https://www.conoe.it/chi-siamo-2/.

[13] Ibrahim S.M.A., Abed K.A., Gad M.S., Abu Hashish H.M. (2023). A semi-industrial reactor for producing biodiesel from waste cooking oil. Biofuels.

[14] Biundo A., Stamm A., Gorgoglione R., Syrén P.O., Curia S., Hauer B., Capriati V., Vitale P., Perna F., Agrimi G. (2023). Regio- and stereoselective biocatalytic hydration of fatty acids from waste cooking oils en route to hydroxy fatty acids and bio-based polyesters. Enzyme Microb. Technol..

[15] Sole R., Buranello C., Di Michele A., Beghetto V. (2022). Boosting physical-mechanical properties of adipic acid/chitosan films by DMTMM cross-linking. Int. J. Biol. Macromol..

[16] Cayzer S., Griffiths P., Beghetto V. (2017). Design of indicators for measuring product performance in the circular economy. Int. J. Sustain. Eng..

[17] Beghetto V., Sole R., Buranello C., Al-Abkal M., Facchin M. (2021). Recent advancements in plastic packaging recycling: A mini-review. Materials.

[18] Kümmerer K., Clark J.H., Zuin V.G. (2020). Rethinking chemistry for a circular economy. Science.

[19] Beghetto V., Gatto V., Samiolo R., Scolaro C., Brahimi S., Facchin M., Visco A. (2023). Plastics today: Key challenges and EU strategies towards carbon neutrality: A review. Environ. Pollut..

[20] Scrivanti A., Sole R., Bortoluzzi R., Beghetto V., Bardella N., Dolmella A. (2019). Synthesis of new triazolyl-oxazoline chiral ligands and study of their coordination to Pd(II) metal centers. Inorg. Chim. Acta.

[21] Uz V.E., Gökalp I. (2020). Sustainable recovery of waste vegetable cooking oil and aged bitumen: Optimized modification for short and long term aging cases. Waste Manag..

[22] Xu N., Wang H., Chen Y., Hossiney N., Ma Z., Wang H. (2023). Insight into the effects of waste vegetable oil on self-healing behavior of bitumen binder. Constr. Build. Mater..

[23] Sun Z., Yi J., Huang Y., Feng D., Guo C. (2016). Properties of asphalt binder modified by bio-oil derived from waste cooking oil. Constr. Build. Mater..

[24] Asli H., Ahmadinia E., Zargar M., Karim M.R. (2012). Investigation on physical properties of waste cooking oil—Rejuvenated bitumen binder. Constr. Build. Mater..

[25] Wan Azahar W.N.A., Bujang M., Ramadhansyah P.J., Hainin M.R., Ngadi N., Al Bakri Abdullah M.M. (2016). Performance of waste cooking oil in asphalt binder modification. Key Eng. Mater..

[26] Oldham D., Rajib A., Dandamudi K.P.R., Liu Y., Deng S., Fini E.H. (2021). Transesterification of waste cooking oil to produce a sustainable rejuvenator for aged asphalt. Resour. Conserv. Recycl..

[27] Wan Azahar W.N.A., Jaya R.P., Hainin M.R., Bujang M., Ngadi N. (2017). Mechanical performance of asphaltic concrete incorporating untreated and treated waste cooking oil. Constr. Build. Mater..

[28] Vyas A.P., Verma J.L., Subrahmanyam N. (2010). A review on FAME production processes. Fuel.

[29] Boiling Point Determination. https://www.jove.com/it/science-education/11192/determination-of-boiling-points-by-capillary-method-concept.

[30] Miskah S., Aprianti T., Agustien M., Utama Y., Said M. (2019). Purification of used cooking oil using activated carbon adsorbent from durian peel. IOP Conf. Ser. Earth Environ. Sci..

[31] Matteoli U., Beghetto V., Scrivanti A., Aversa M., Bertoldini M., Bovo S. (2011). An alternative stereoselective synthesis of (R)- and (S)-Rosaphen^®^ via Asymmetric Catalytic Hydrogenation. Chirality.

[32] Dean E.W., Stark D.D. (1920). A convenient method for the determination of water in petroleum and other organic emulsions. J. Ind. Eng. Chem..

[33] Yadykova A.Y., Strelets L.A., Ilyn S.O. (2023). Infrared spectral classification of natural bitumens for their rheological and thermophysical characterization. Molecules.

[34] Yang F., Duan Z., Liu D., Li C., Sun G., Zhang H. (2022). Multi-alkylated aromatic amides amphiphiles effectively stabilize the associated asphaltene particles in crude oil. J. Pet. Sci. Eng..

[35] Mendelez-Alvarez A.A., Garcia-Bermudes M., Tavakkoli M., Doherty R.H., Meng S., Abdallah D.S., Vargas F.M. (2016). On the evaluation of the performance of asphaltene dispersants. Fuel.

[36] Guzmán R., Ancheyta J., Trejo F., Rodríguez S. (2017). Methods for determining asphaltene stability in crude oils. Fuel.

[37] Heithaus J. (1962). Measurement and significance of asphaltene peptization. J. Inst. Petrol.

[38] Schermer W.E.M., Melein P.M.J., Van den Berg F.G.A. (2004). Simple techniques for evaluation of crude oil compatibility. Petrol Sci. Technol..

[39] Ujong A.E., Emelike N.J.T., Owuno F., Okiyi P.N. (2023). Effect of frying cycles on the physical, chemical and antioxidant properties of selected plant oils during deep-fat frying of potato chips. Food Chem. Adv..

[40] Niyas M.M., Shaija A. (2023). Effect of fatty acid profiles of waste cooking oil biodiesels on their thermal and physical properties. J. Therm. Anal. Calorim..

[41] Chuah L.F., Klemeš J.J., Yusup S., Bokhari A., Akbar M.M. (2017). Influence of fatty acids in waste cooking oil for cleaner biodiesel. Clean. Technol. Environ. Policy.

[42] Putraa R.S., Juliantoa T.S., Hartonoa P., Puspitasaria R.D., Kurniawan A. (2014). Pre-treatment of Used-Cooking Oil as Feed Stocks of Biodiesel Production by Using Activated Carbon and Clay Minerals. Int. J. Renew. Energy Dev..

[43] Rahayu S., Supriyatin S., Bintari A. (2018). Activated carbon-based bio-adsorbent for reducing free fatty acid number of cooking oil. AIP Conf. Proc..

[44] Wan Azahar W.N.A., Ramadhansyah P.J., Hainin M.R., Bujang M., Ngadi N. (2016). Chemical modification of waste cooking oil to improve the physical and rheological properties of asphalt binder. Constr. Build. Mater..

[45] Rahayu S., Pambudi K.A., Afifah A., Fitriani S.R., Tasyari S., Zaki M., Djamahar R. (2021). Environmentally safe technology with the conversion of used cooking oil into soap. J. Phys. Conf. Ser..

[46] Allen C.L., Chhatwala A.R., Williams J.M.J. (2012). Direct amide formation from unactivated carboxylic acids and amines. Chem. Commun..

[47] Smith M.E., Adkins H. (1938). The relative reactivity of amines in the aminolysis of amides. J. Am. Chem. Soc..

[48] Sole R., Gatto V., Conca S., Bardella N., Morandini A., Beghetto V. (2021). Sustainable triazine-based dehydro-condensation agents for amide synthesis. Molecules.

[49] Liu Y., Su P., Li M., You Z., Zhao M. (2020). Review on evolution and evaluation of asphalt pavement structures and materials. J. Traffic Transp. Eng..

[50] Beghetto V., Bardella N., Samiolo R., Gatto V., Conca S., Sole R., Molin G., Gattolin A., Ongaro N. (2021). By-products from mechanical recycling of polyolefins improve hot mix asphalt performance. J. Clean. Prod..

[51] Jain S., Singh B. (2021). Cold mix asphalt: An overview. J. Clean. Prod..

[52] United Nations Transforming Our World: The 2030 Agenda for Sustainable Development. https://sustainabledevelopment.un.org/post2015/transformingourworld/publication.

[53] Hidalgo A.E., Moreno-Navarro F., Tauste R., Rubio-Gámez M.C. (2020). The Influence of Reclaimed Asphalt Pavement on the Mechanical Performance of Bituminous Mixtures. An Analysis at the Mortar Scale. Sustainability.

[54] Milad A., Taib A.M., Ahmeda A.G.F., Solla M., Yusoff N.I.M. (2020). A review of the use of reclaimed asphalt pavement for road paving applications. J. Teknol..

[55] Babashamsi P., Yusoff N.I.M., Ceylan H., Ghani N., Nor M. (2016). Recycling toward sustainable pavement development: End-of-life considerations in asphalt pavement. J. Teknol..

[56] Tarsi G., Tataranni P., Sangiorgi C. (2020). The challenges of using reclaimed asphalt pavement for new asphalt mixtures: A review. Materials.

[57] Sakthivel S.N., Kathuria A., Singh B. (2022). Utilization of inferior quality aggregates in asphalt mixes: A systematic review. J. Traffic Transp. Eng..

[58] Lesuer D. (2009). The colloidal structure of bitumen: Consequences on the rheology and on the mechanisms of bitumen modification. Adv. Colloid Interface Sci..

[59] Holý M., Remišová E. (2019). Analysis of influence of bitumen composition on the properties represented by empirical and viscosity test. Transp. Res. Procedia.

[60] Loise V., Caputo P., Porto M., Calandra P., Angelico R., Rossi C.O. (2019). A review on bitumen rejuvenation: Mechanisms, materials, methods and perspectives. Appl. Sci..

[61] Fini E.H., Buabeng F.S., Abu-Lebdeh T., Awadallah F. (2016). Effect of introduction of furfural on asphalt binder ageing characteristics. Road Mater. Pavement Des..

[62] Pahlavan F., Rajib A., Deng S., Lammers P., Fini E.H. (2020). Investigation of balanced feedstocks of lipids and proteins to synthesize highly effective rejuvenators for oxidized asphalt. ACS Sustain. Chem. Eng..

[63] Loise V., Calandra P., Abe A.A., Porto M., Rossi C.O., Davoli M., Caputo P. (2021). Additives on aged bitumen: What probe to distinguish between rejuvenating and fluxing effects?. J. Mol. Liq..

[64] (2018). Standard Test Method for Separation of Asphalt into Four Fractions.

[65] Prosperi E., Bocci E. (2021). A review on bitumen aging and rejuvenation chemistry: Processes, materials and analyses. Sustainability.

[66] Remišová E., Holý M. (2017). Changes of properties of bitumen binders by additives application. IOP Conf. Ser. Mater. Sci. Eng..

