# Supplementary material for: Waste Cooking Oil as Eco-Friendly Rejuvenator for Reclaimed Asphalt Pavement
Source: Materials (Basel). 2024 Mar 24;17(7):1477. doi: 10.3390/ma17071477 (PMC11012562; doi:10.3390/ma17071477)
Supplement: Supplementary file 1 [file materials-17-01477-s001.zip › materials-2918443-supplementary.pdf]

Waste Cooking Oil as Eco-Friendly Rejuvenator for Reclaimed Asphalt Pavement

Supplementary Materials

Noemi Bardella [1], Manuela Facchin [2], Eleonora Fabris [2], Matteo Baldan [2] and Valentina Beghetto [1,2,3,*]

[1] Crossing S.r.l., Viale della Repubblica 193/b, 31100 Treviso, Italy; noemi.bardella@crossing-srl.com
[2] Department of Molecular Sciences and Nanosystems, University Ca' Foscari of Venice, Via Torino 155, 30172 Venice, Italy; matteo.baldan@unive.it (M.B.)
[3] Consorzio Interuniversitario per le Reattività Chimiche e la Catalisi (CIRCC), Via C. Ulpiani 27, 70126 Bari, Italy
* Correspondence: beghetto@unive.it; Tel.: +39-0412348928

Content:

1) ^{1}H, ^{13}C NMR of purified WCO
2) ^{1}H, ^{13}C NMR of purified hydrolyzed WCO (HWCO)
3) ^{1}H, ^{13}C NMR of additives I-XII
4) Calculation of the Heithaus parameter for bitumen and for bitumen added with 25 wt% of additive (VII) by weight of bitumen.

1) NMR of purified WCO

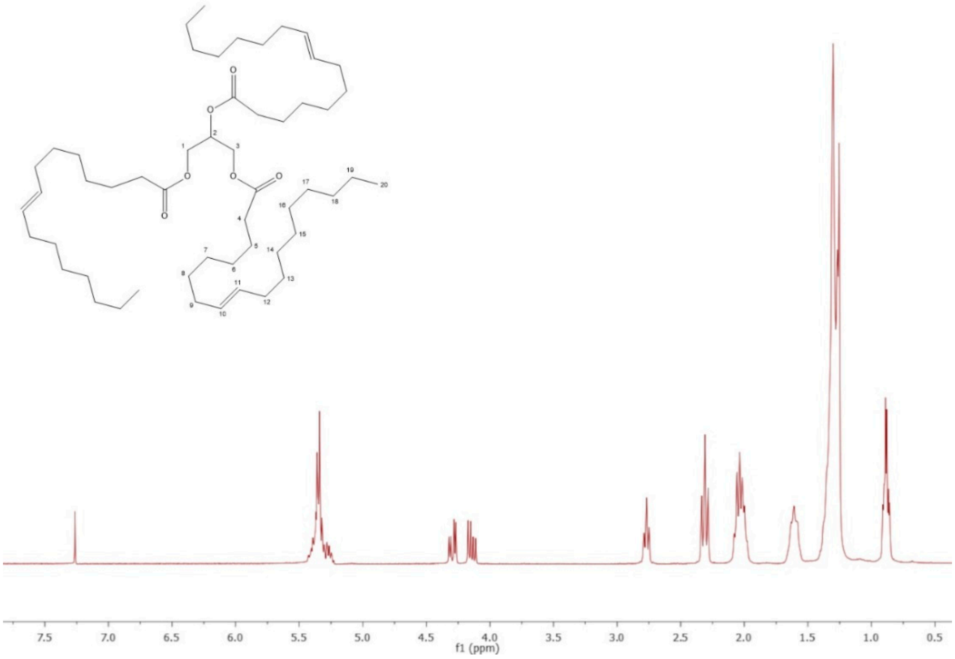

Figure S1. ^{1}H NMR of purified WCO
δ (ppm) (300 MHz, $CDCl_3$): 5.33 (6H, m, H_{10-11}), 5.26 (1H, m, H_2), 4.30 - 4.10 (4H, m, H_1 H_3), 2.30 (6H, t, H_4), 2.01 (12H, m, H_9 H_{12}), 1.60 (6H, m, H_5), 1.29 (72H, m, H_{6-7} H_{13-19}), 0.87 (6H, t, H_{20}).

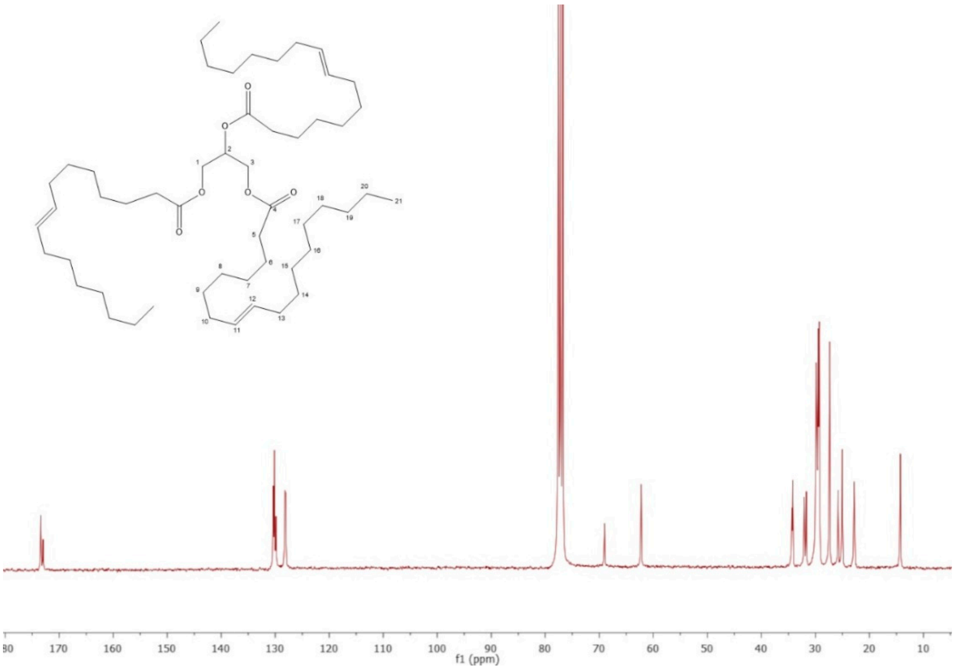

Figure S2. ^{13}C NMR of purified WCO
δ (ppm) (400 MHz, $CDCl_3$): 173.31 (C4), 130.24 (C_{11-12}), 68.89 (C_2), 61.88 (C_1 C_3), 34.08 (C_5), from 29.77 to 29.05 (C_{7-9} C_{14-20}), 27.21 (C_{10} C_{13}), 25.65 (C_6), 14.11 (C_{21})

2) NMR of HWCO

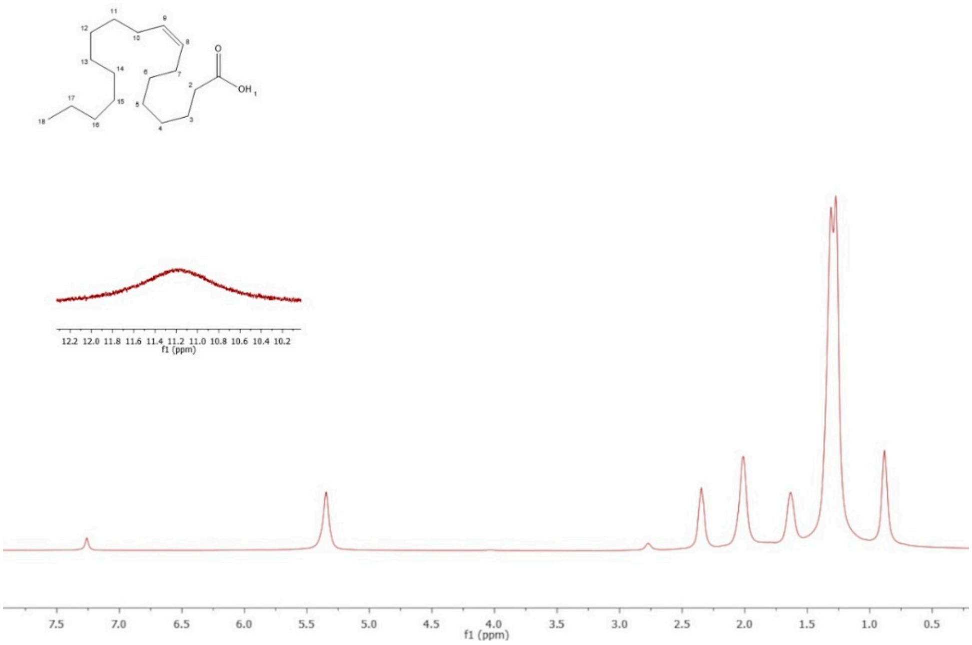

Figure S3. ^{1}H NMR of hydrolysed WCO (HWCO)
δ (ppm) (400 MHz, $CDCl_3$): 11.24 (1H, s, H_1), 5.36 (2H, m, H_{8-9}), 2.34 (2H, t, H_2), 2.02 (4H, m, H_7 H_{10}), 1.64 (2H, m, H_3), 1.30 (20H, m, H_{4-6} , H_{11-17}), 0.88 (3H, t, H_{18})

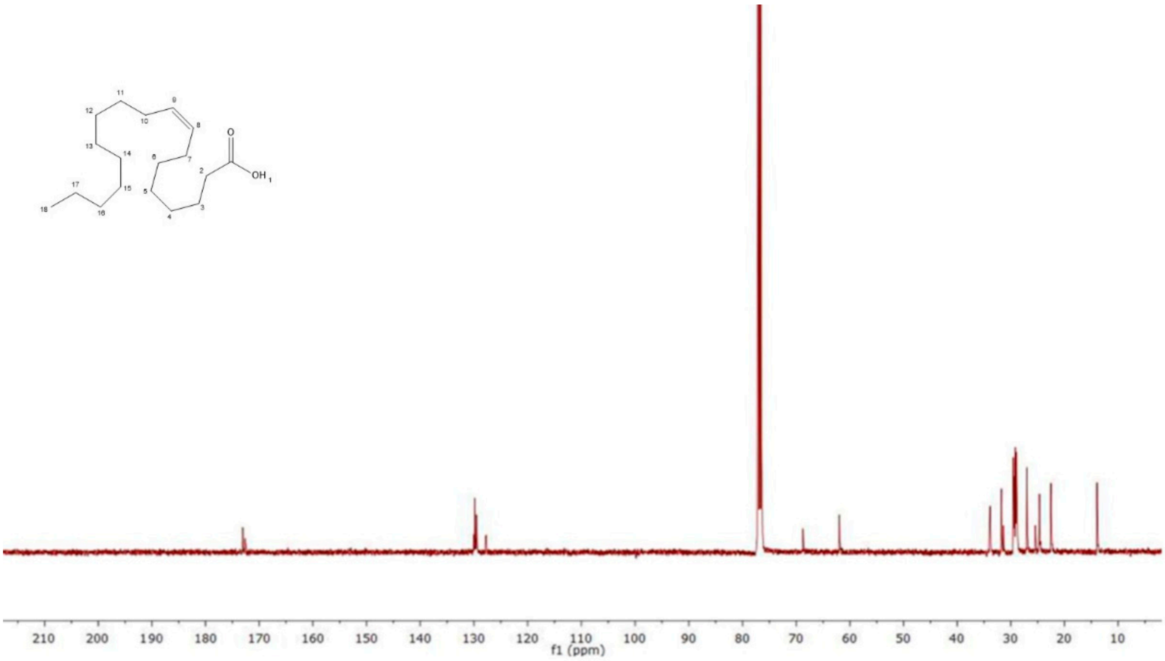

Figure S4. ^{13}C NMR of hydrolysed WCO (HWCO)
δ (ppm) (101 MHz, $CDCl_3$): 180.61 (C_1), 129.94 (C_{8-9}), 34.33 (C_2), from 31.94 to 29.07 (C_{4-6} C_{11-17}), 27.23 (C_7 C_{10}), 24.67 (C_3), 14.07 (C_{18}).

3) NMR of additives I-XII

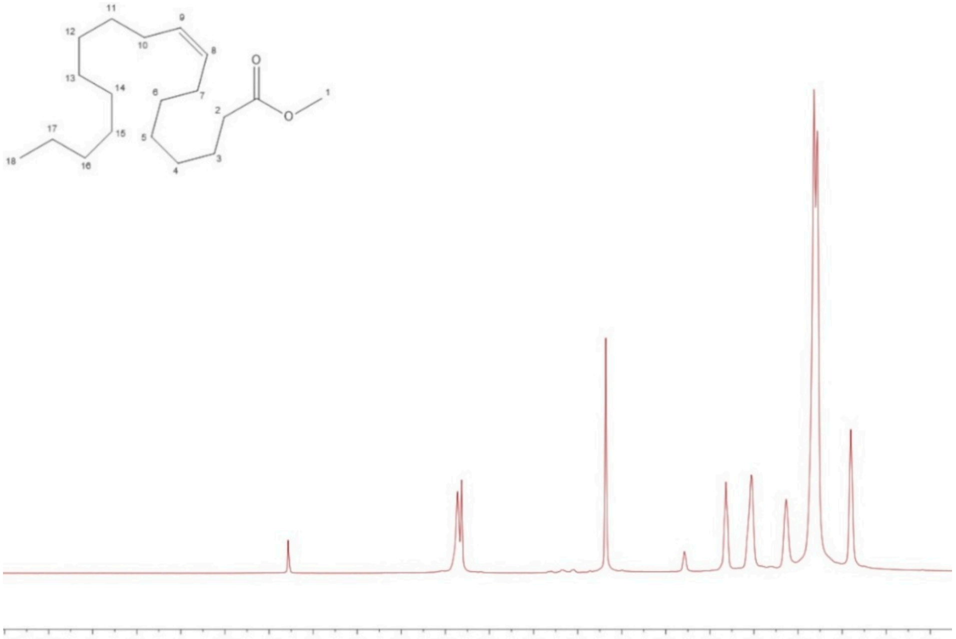

Figure S5. ^{1}H NMR of additive I
δ (ppm) (400 MHz, $CDCl_3$): 5.36 (2H, m, H_{8-9}), 3.66 (3H, s, H_1), 2.31 (2H, t, H_2), 2.02 (4H, m, H_7 H_{10}), 1.61 (2H, m, H_3), 1.30 (22H, m, H_{4-6} H_{10-17}), 0.88 (3H, t, H_{18}).

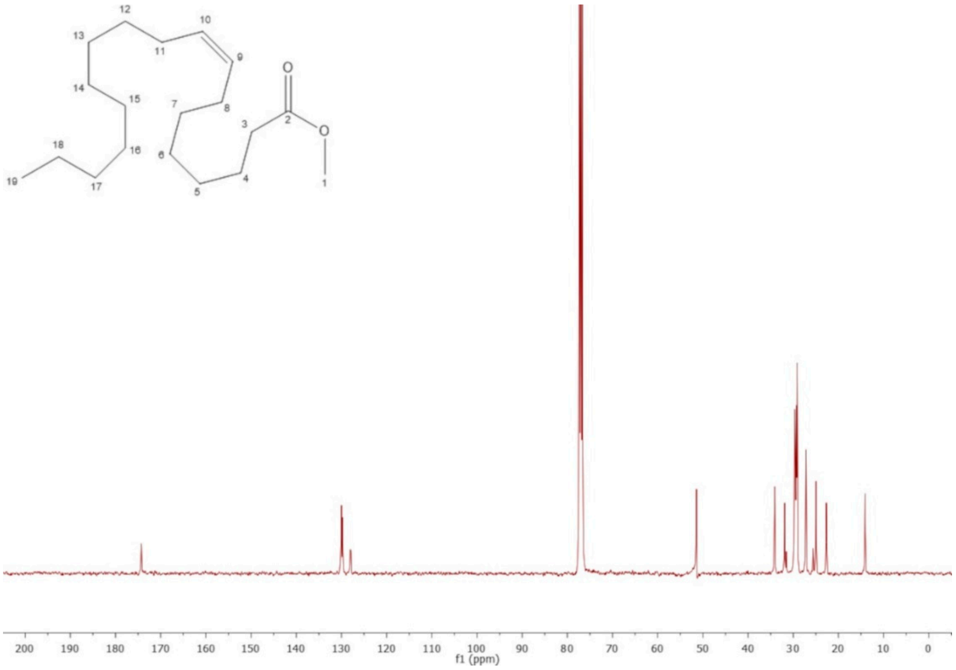

Figure S6. ^{13}C NMR of additive I
δ (ppm) (101 MHz, $CDCl_3$): 174.43 (C_2), 130.15 (C_{9-10}), 53.56 (C_1), 34.26 (C_3), from 32.06 to 29.47 (C_{6-7} C_{12-18}), 27.37 (C_8 C_{11}), 25.11 (C_4), 14.26 (C_{19}).

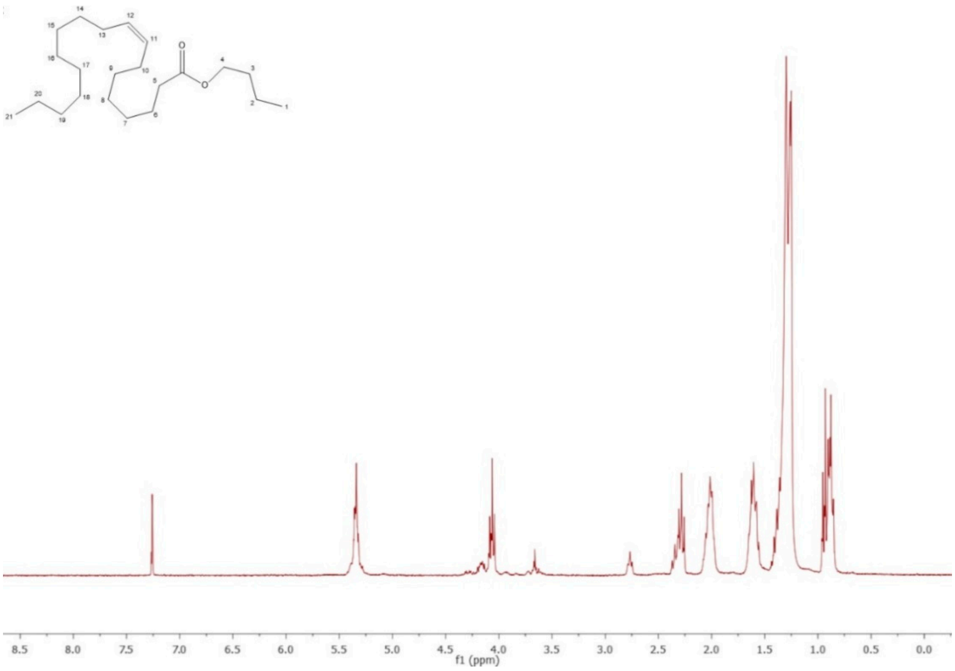

Figure S7. ^{1}H NMR of additive II

δ (ppm) (400 MHz, $CDCl_3$): 5.36 (2H, m, H_{11-12}), 4.06 (2H, t, H_4), 2.28 (2H, t, H_5), 2.09 (4H, m, H_{10} H_{13}), 1.61 (4H, m, H_3 H_6), 1.30 (22H, m, H_2 H_{7-9} H_{14-20}), 0.88 (6H, m, H_1 H_{21}).

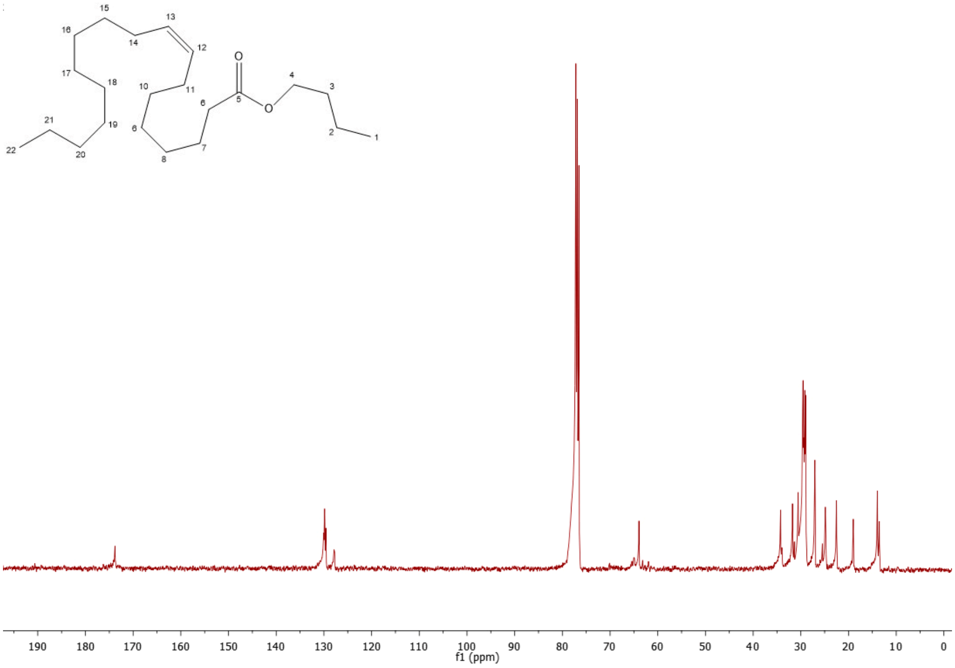

Figure S8. ^{13}C NMR of additive II

δ (ppm) (101 MHz, $CDCl_3$): 173.81 (C_5), 129.86 (C_{12-13}), 63.93 (C_4), 34.23 (C_6), from 30.55 to 28.96 (C_{2-3} C_{8-10} C_{15-21}), 27.05 (C_{11} C_{14}), 24.85 (C_7), 13.93 (C_1 C_{22}).

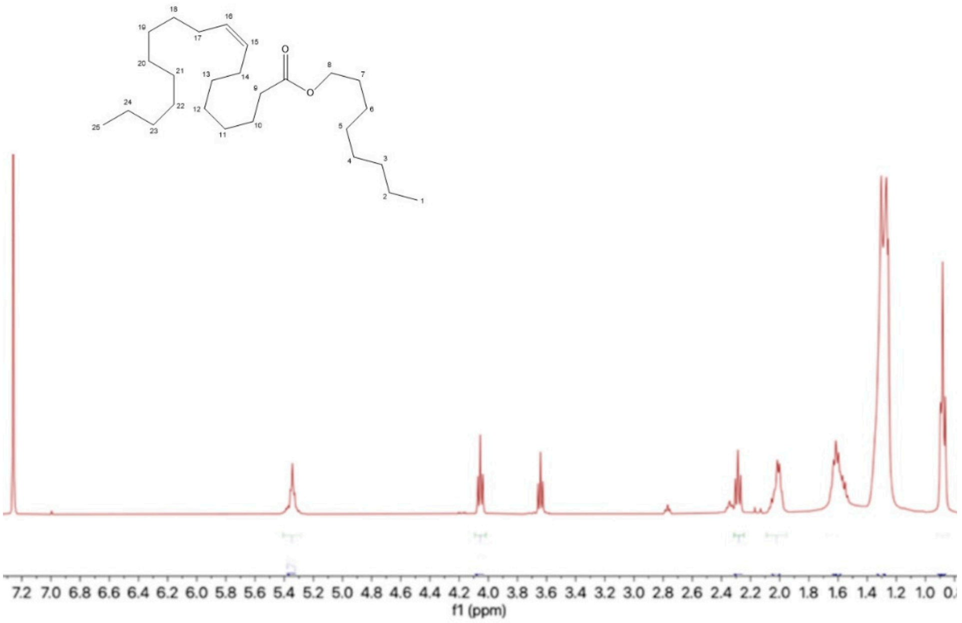

Figure S9. ^{1}H NMR of additive III
δ (ppm) (400 MHz, $CDCl_3$): 5.34 (2H, m, H_{15-16}), 4.05 (2H, t, H_8), 2.29 (2H, t, H_9), 2.02 (4H, m, H_{14} H_{17}), 1.62 (6H, m, H_{6-7} H_{10}), 1.30 (28H, m, H_{2-5} H_{11-13} H_{18-24}), 0.88 (6H, m, H_1, H_{25}).

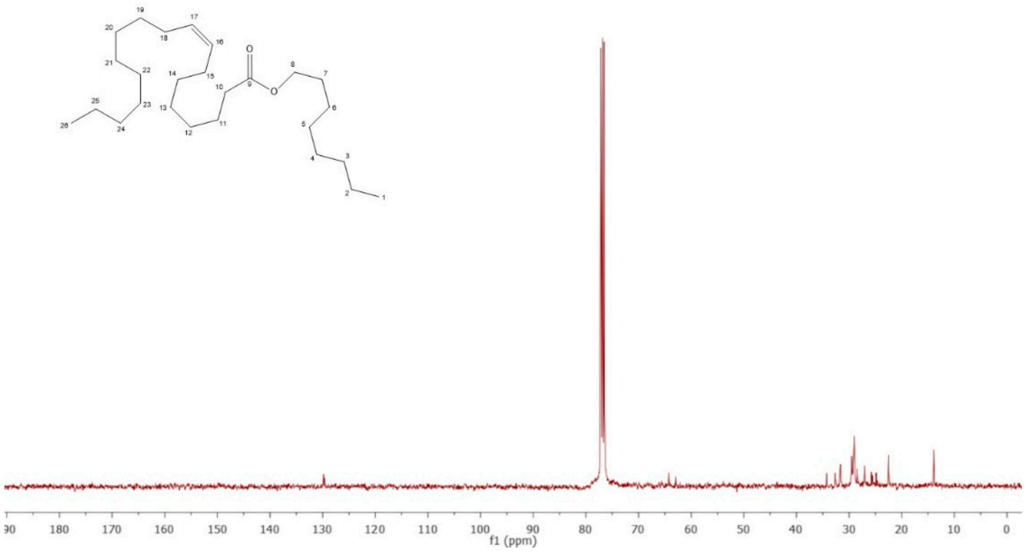

Figure S10. ^{13}C NMR of additive III
δ (ppm) (101 MHz, $CDCl_3$): 174.15 (C_9), 130.15 (C_{16-17}), 64.56 (C_8), 34.57 (C_{10}), from 32.97 to 28.82 (C_{2-7} C_{12-14} C_{19-25}), 26.09 (C_{15} C_{18}), 25.18 (C_{11}), 14.23 (C_1 C_{26}).

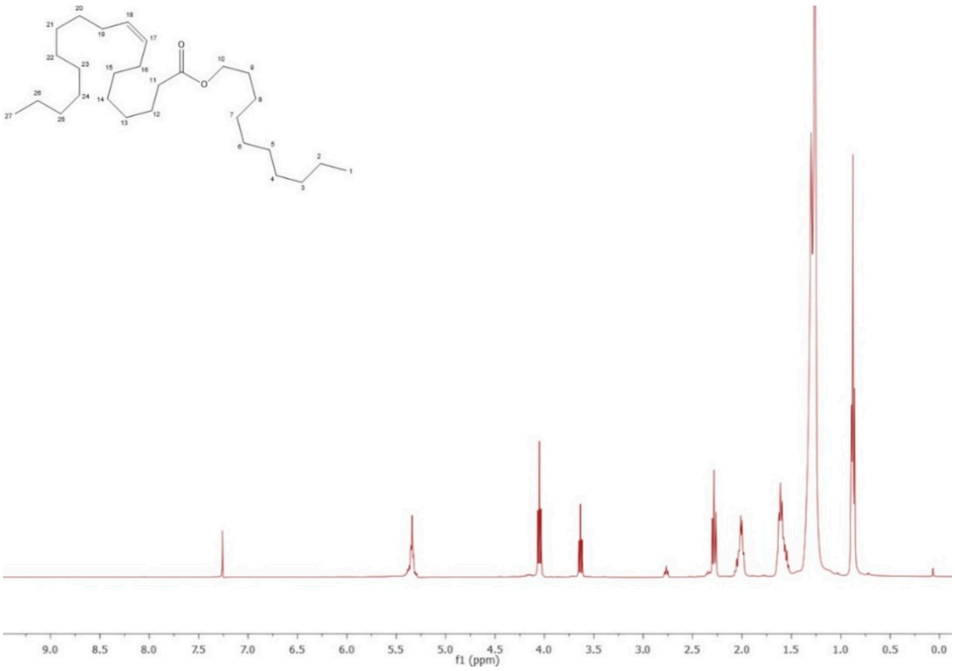

Figure S11. ^{1}H NMR of additive IV

δ (ppm) (400 MHz, $CDCl_3$): 5.34 (2H, m, H_{17-18}), 4.05 (2H, t, H_{10}), 2.29 (2H, t, H_{11}), 2.00 (4H, m, H_{16} H_{19}), 1.61 (4H, m, H_{9} H_{12}), 1.27 (34H, m, H_{2-8} H_{13-15} H_{20-26}), 0.88 (6H, m, H_{1} H_{27}).

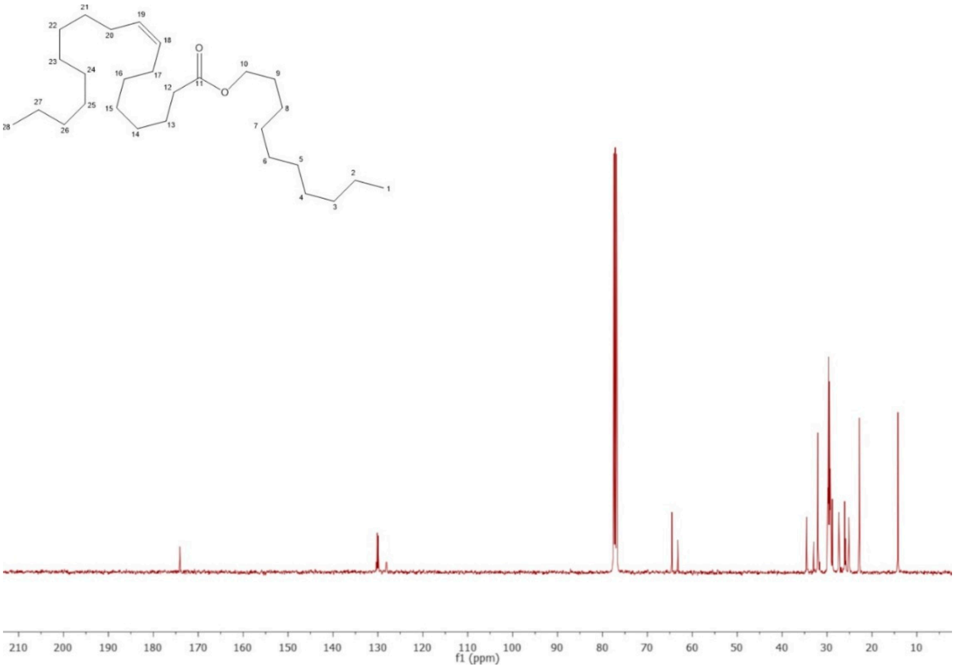

Figure S12. ^{13}C NMR of additive IV

δ (ppm) (101 MHz, $CDCl_3$): 174.11 (C_{11}), 130.16 (C_{18-19}), 64.55 (C_{10}), 34.56 (C_{12}), from 32.04 to 28.81 (C_{2-9} C_{14-16} C_{21-27}), 27.05 (C_{17} C_{20}), 24.85 (C_{13}), 13.93 (C_{1} C_{28}).

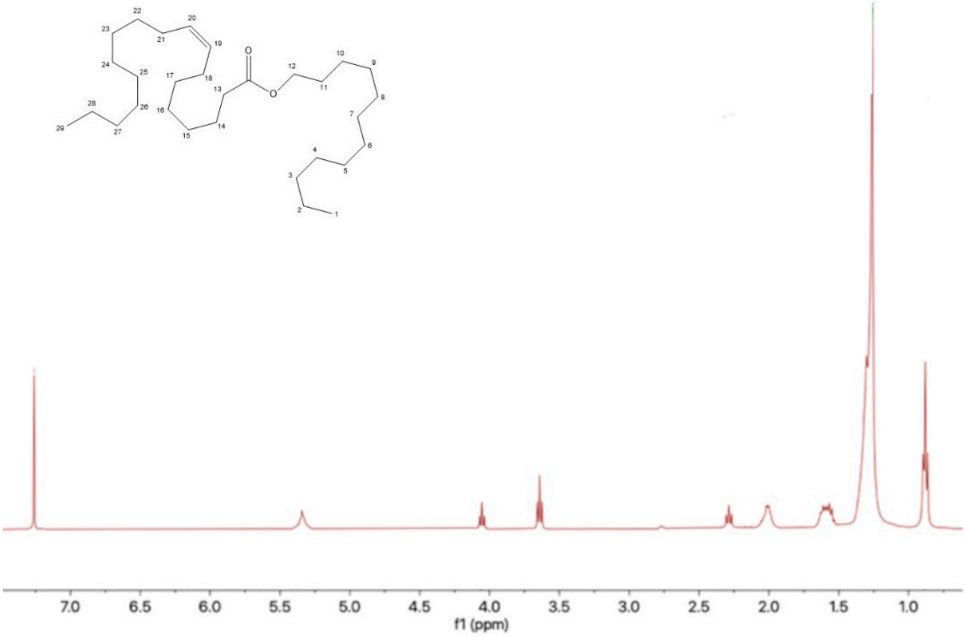

Figure S13. ^{1}H NMR of additive V

δ (ppm) (400 MHz, $CDCl_3$): 5.34 (2H, m, H_{19-20}), 4.05 (2H, t, H_{12}), 2.29 (2H, t, H_{13}), 2.00 (4H, m, H_{18} H_{21}), 1.59 (4H, m, H_{11} H_{14}), 1.26 (38H, m, H_{2-10} H_{15-17} H_{22-28}), 0.88 (6H, m, H_1 H_{29}).

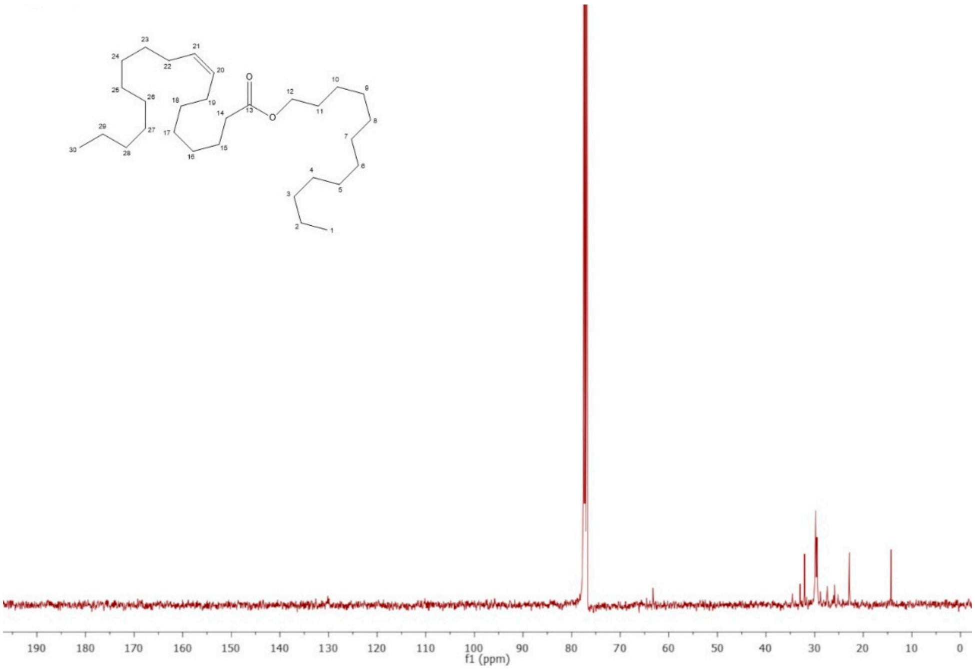

Figure S14. ^{13}C NMR of additive V

δ(ppm) (101 MHz, $CDCl_3$): 175.40 (C_{13}), 130.15 (C_{20-21}), 64.56 (C_{12}), 34.57 (C_{14}), 32.97 (C_{19}, C_{22}), 32.07 (C_{11}), 29.79 (C_{4-9} C_{15-18} C_{23-27} C_{25-30}), 28.82 (C_{10}), 27.38 (C_{15}), 25.90 (C_8), 25.18 (C_{28}), 22.83 (C_2, C_{29}), 14.26 (C_1, C_{30}).

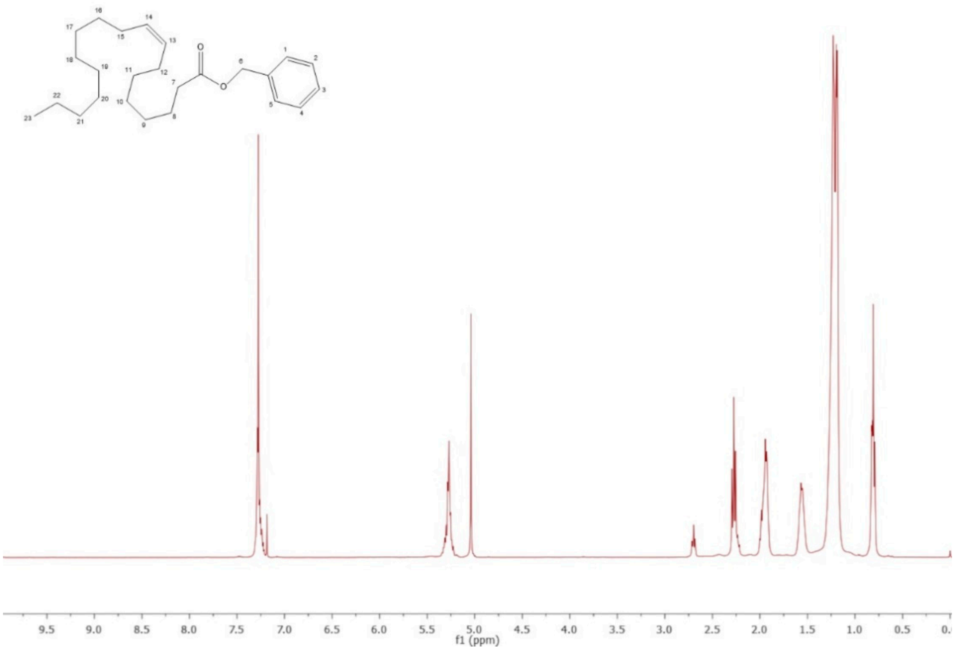

Figure S15. ^{1}H NMR of additive V

δ (ppm) (400 MHz, $CDCl_3$): 7.45-7.30 (5H, m, H_{1-5}), 5.34 (2H, m, H_{13-14}), 5.11 (2H, s, H_6), 2.35 (2H, t, H_7), 2.02 (4H, m, H_{12} H_{15}), 1.64 (2H, m, H_8), 1.26 (20H, m, H_{9-11} H_{16-22}), 0.88 (3H, t, H_{23}).

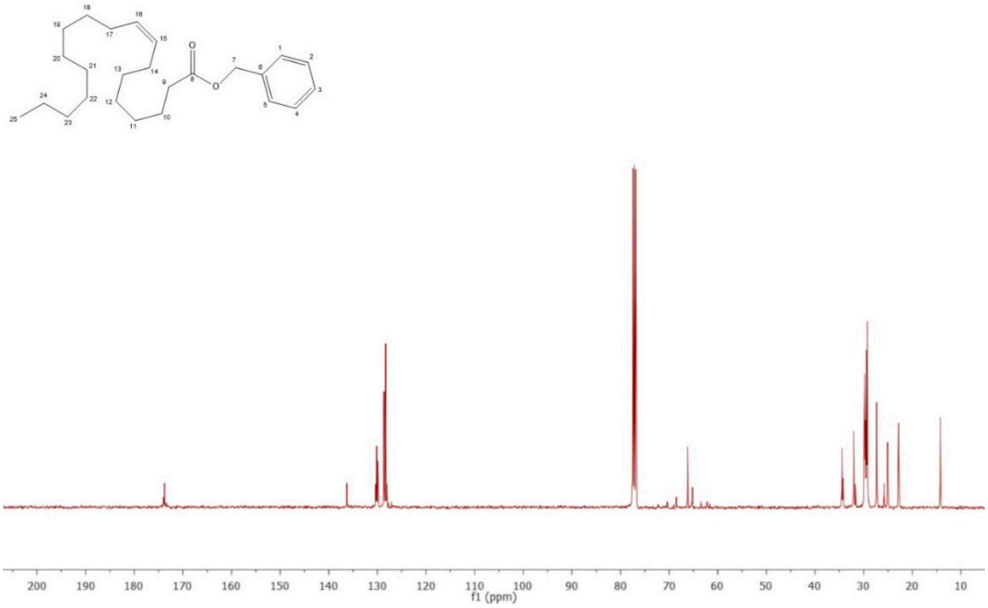

Figure S16. ^{13}C NMR of additive VI

δ (ppm) (101 MHz, $CDCl_3$): 173.78 (C_8), 136.28 (C_6), 130.17 (C_{15-16}), from 128.66 to 128.88 (C_{1-5}), 66.19 (C_7), 34.47 (C_9), from 32.04 to 29.23 (C_{11-13} C_{18-24}), 27.36 (C_{14} C_{17}), 25.09 (C_{10}), 14.25 (C_{25}).

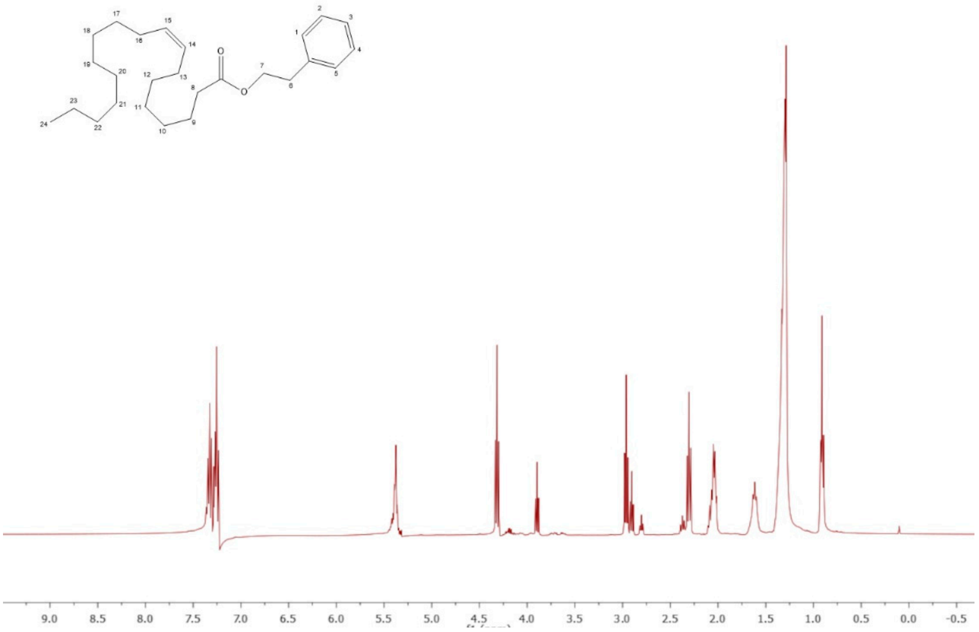

Figure S17. ^{1}H NMR of additive VII

δ (ppm) (400 MHz, $CDCl_3$): 7.45-7.30 (5H, m, H_{1-5}), 5.38 (2H, m, H_{14-15}), 4.32 (2H, t, H_7), 2.91 (2H, m, H_6), 2.31(2H, t, H_8), 2.05 (4H, m, H_{13} H_{16}), 1.62 (2H, m, H_9), 1.30 (20H, m, H_{10-12} H_{17-23}), 0.92 (3H, t, H_{24}).

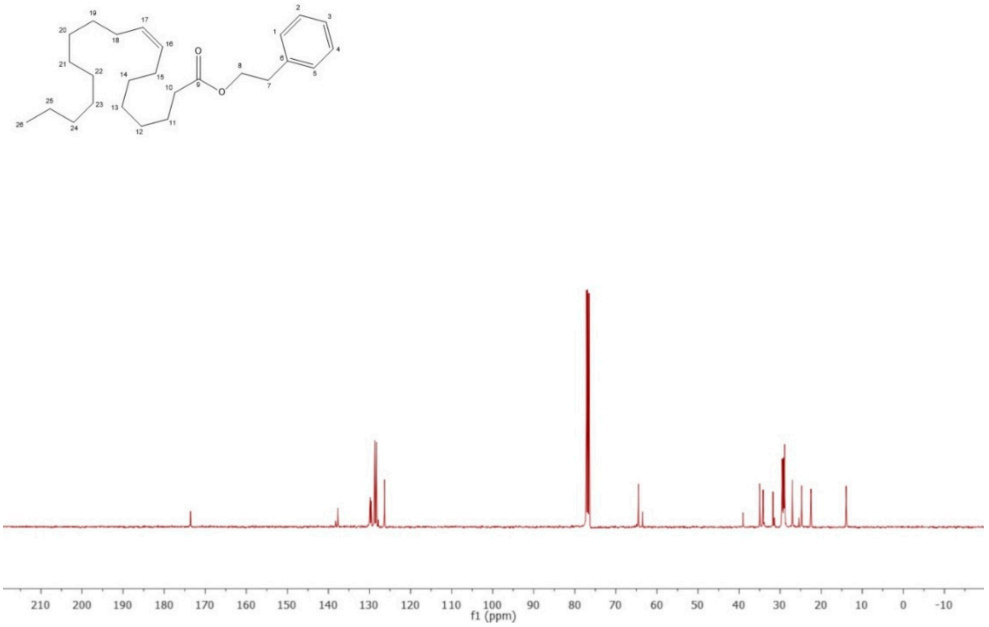

Figure S18. ^{13}C NMR of additive VII

δ (ppm) (101 MHz, $CDCl_3$): 173.91 (C_9), 138.02 (C_6), 130.13 (C_{16-17}), from 126.65 to 129.02 (C_{1-5}), 64.82 (C_7), 39.35 (C_{10}), from 35.30 to 29.23 (C_{12-14} C_{19-25}), 27.37 (C_{15} C_{18}), 25.07 (C_{11}), 14.25 (C_{26}).

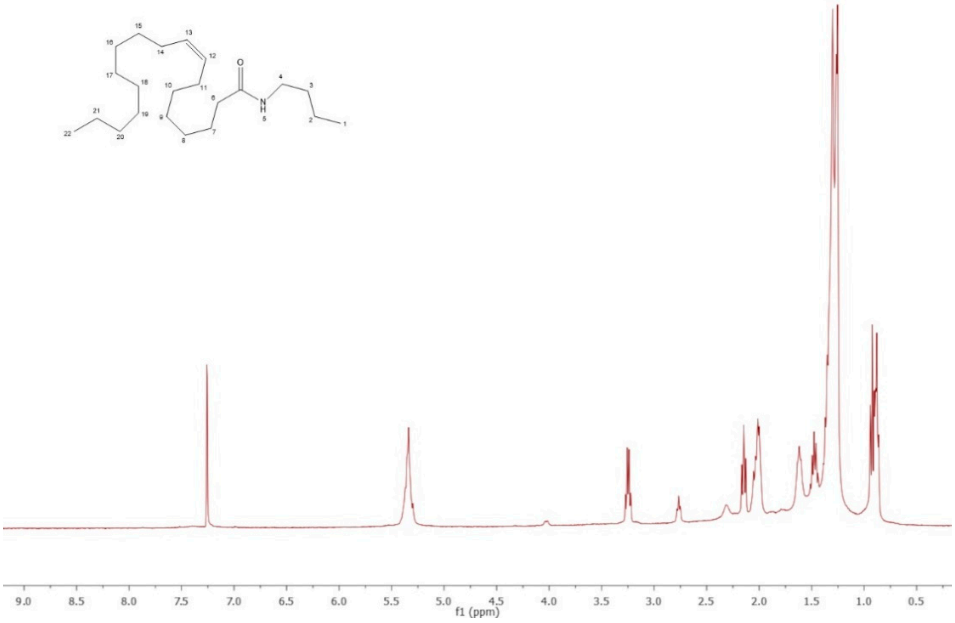

Figure S19. ^{1}H NMR of additive VIII

δ (ppm) (400 MHz, $CDCl_3$): 5.29 (1H, s, H_5), 5.34 (2H, m, H_{12-13}), 3.24 (2H, m, H_4), 2.15 (2H, t, H_6), 2.01 (4H, m, H_{11} H_{14}), 1.62 (2H, m, H_7), 1.48 (2H, m H_3) 1.32 (22H, m, H_2 H_{8-10} H_{15-21}), 0.92 (6H, m, H_1 H_{22}).

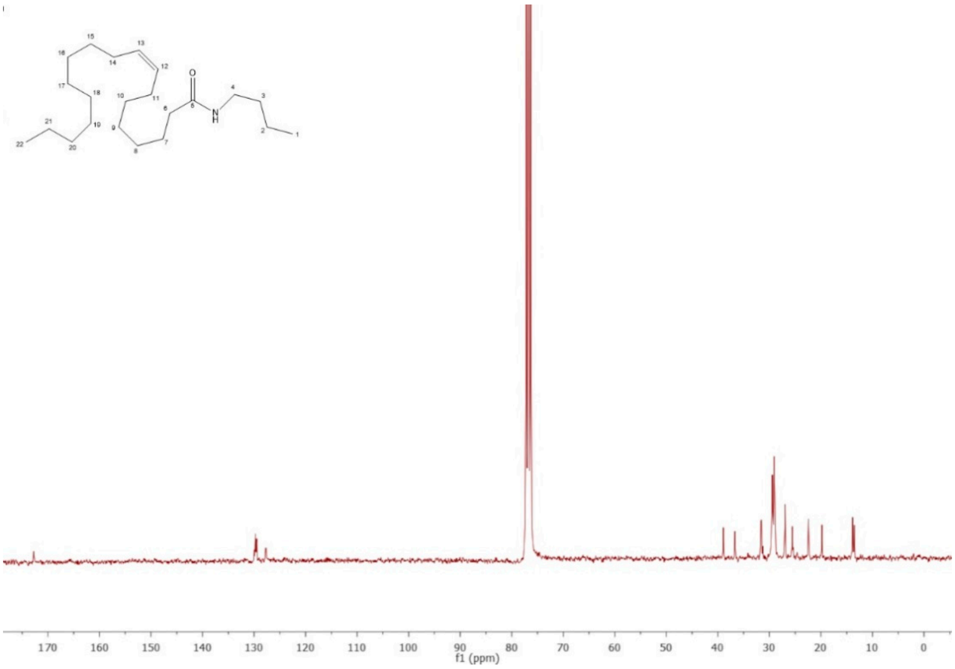

Figure S20. ^{13}C NMR of additive VIII

δ (ppm) (101 MHz, $CDCl_3$): 173.60 (C_5), 130.56 (C_{12-13}), 39.78 (C_4), 37.52 (C_6), from 32.36 to 29.90 (C_2 C_{8-10} C_{15-21}), 26.41 (C_{11} C_{14}), 23.26 (C_7), 14.33 (C_1 C_{22}).

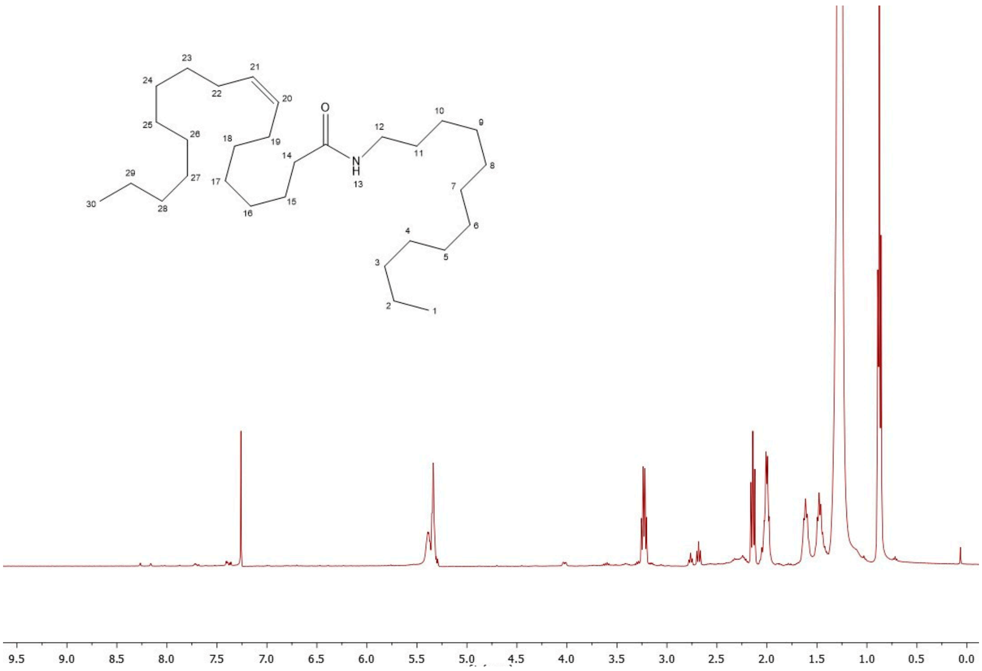

Figure S21. ^{1}H NMR of additive IX

δ (ppm) (400 MHz, $CDCl_3$): 5.39 (1H, m, H_{13}), 5.34 (2H, m, H_{20-21}), 3.22 (2H, m, H_{12}), 2.14 (2H, t, H_{14}), 2.01 (4H, m, H_{19} H_{22}), 1.61 (2H, m, H_{15}), 1.48 (2H, m H_{11}), 1.30 (38H, m, H_{2-10} H_{16-18} H_{23-29}), 0.88 (6H, m, H_1 H_{30}).

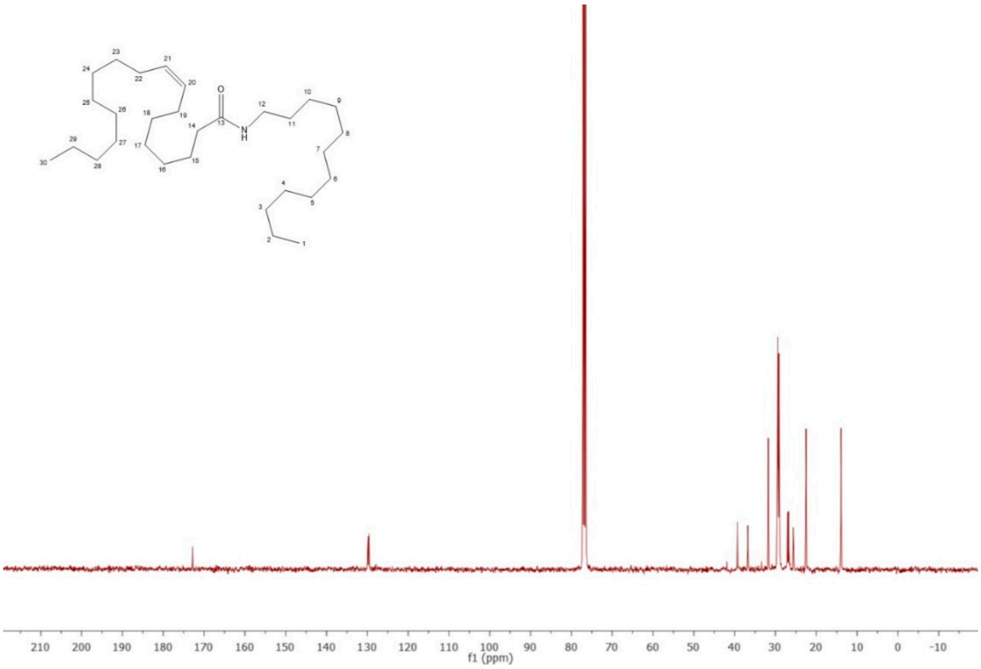

Figure S22. ^{13}C NMR of additive IX

δ (ppm) (101 MHz, $CDCl_3$): 173.11 (C_{13}), 129.89 (C_{20-21}), 39.65 (C_{14}), from 32.06 to 29.78 (C_{2-12} C_{16-18} C_{23-29}), 27.08 (C_{19} C_{22}), 22.83 (C_{15}), 14.25 (C_1 C_{30}).

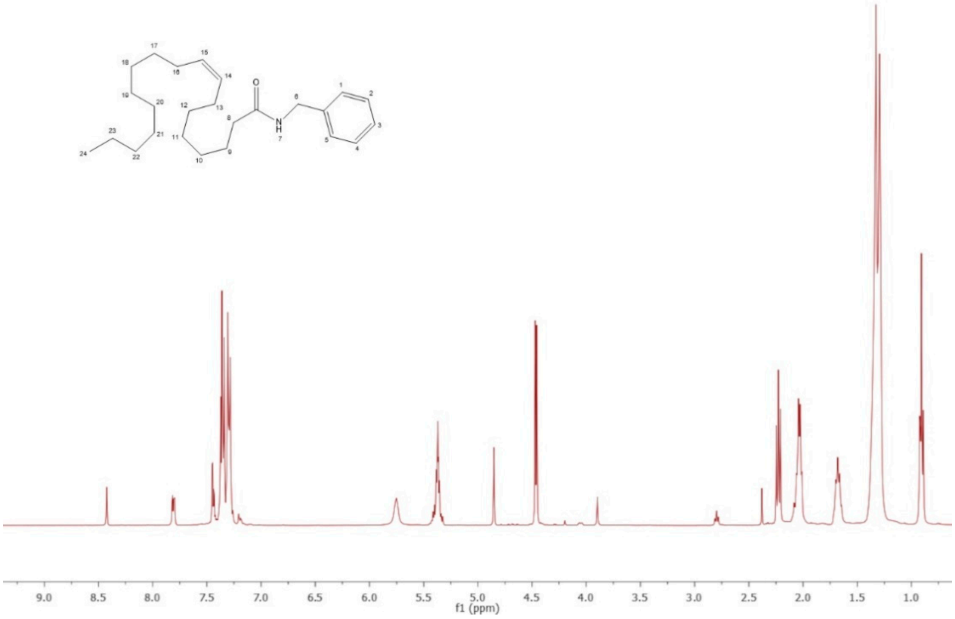

Figure S23. ^{1}H NMR of additive X
δ (ppm) (400 MHz, $CDCl_3$): 7.30 (5H, m $H_{1\text{-}5}$) 5.81 (1H, m, H_7), 5.35 (2H, m, $H_{14\text{-}15}$), 4.81 (2H, d, H_6), 2.18 (2H, t, H_8), 2.00 (4H, m, H_{13} H_{16}), 1.64 (2H, m, H_9), 1.29 (20H, m, $H_{10\text{-}12}$ $H_{17\text{-}23}$), 0.88 (3H, m, H_{24}).

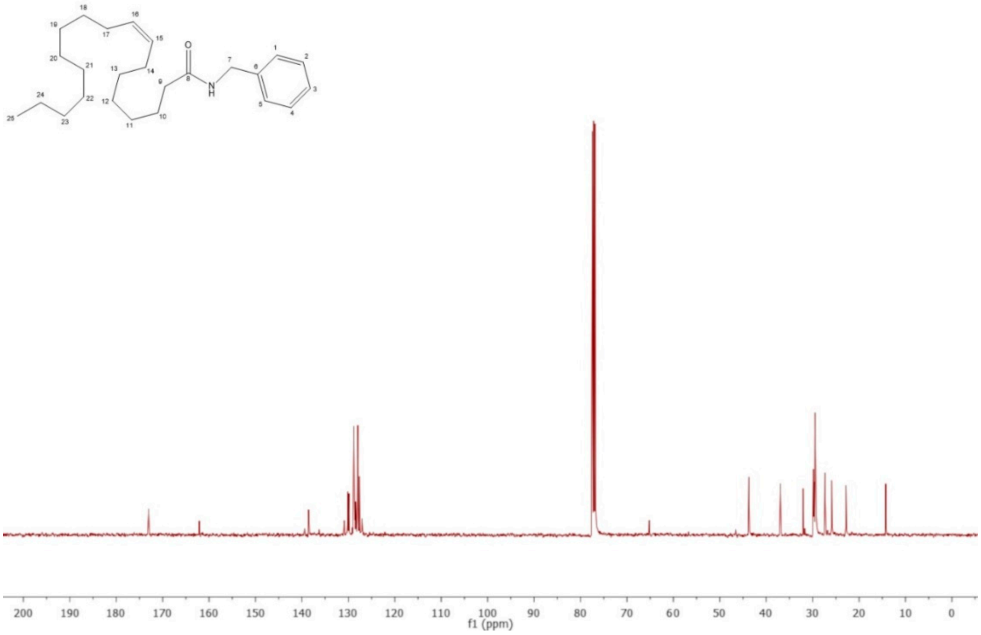

Figure S24. ^{13}C NMR of additive X
δ (ppm) (101 MHz, $CDCl_3$): 173.35 (C_8), 138.90 (C_6), 130.45-130.20 ($C_{15\text{-}16}$), 127.80-127.42 ($C_{1\text{-}5}$), 44.04 (C_7), 37.26 (C_9), from 32.36 to 29.78 ($C_{11\text{-}13}$ $C_{18\text{-}24}$), 27.68 (C_{17}), 26.72 (C_{14}), 23.14 (C_{10}), 14.57 (C_{25})

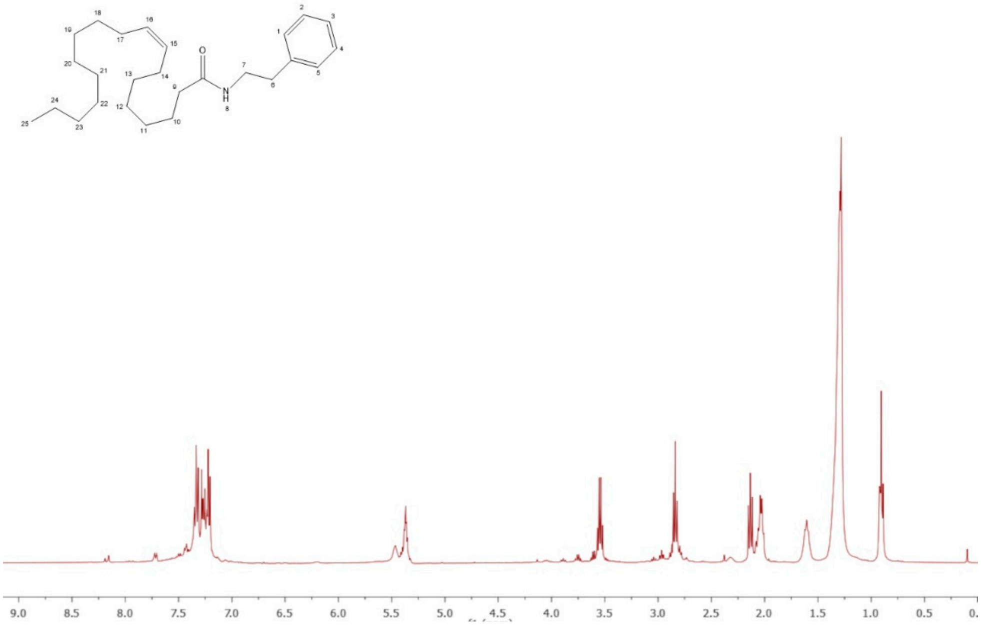

Figure S25. ^{1}H NMR of additive XI

δ (ppm) (400 MHz, $CDCl_3$): 7.30 (5H, m $H_{1\text{-}5}$), 5.35 (2H, m, $H_{15\text{-}16}$), 5.34 (1H, m, H_8), 3.51 (2H, t, H_7), 2.82 (2H, t, H_6), 2.11 (2H, t, H_9), 2.00 (4H, m, H14 H_{17}), 1.58 (2H, m, H_{10}), 1.29 (20H, m, $H_{11\text{-}13}$ $H_{18\text{-}24}$), 0.88 (3H, m, H_{25}).

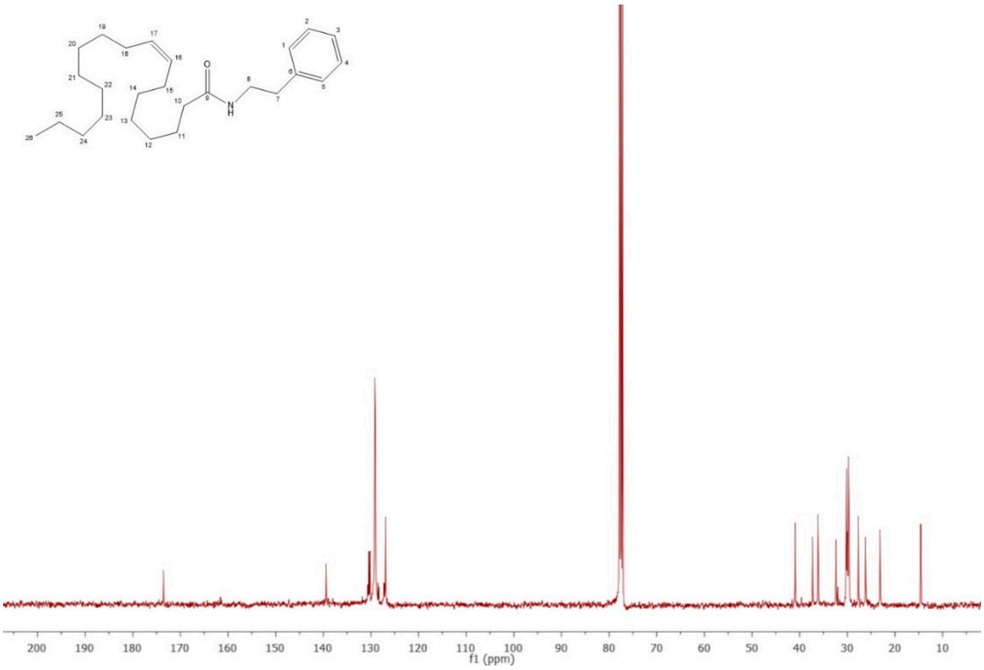

Figure S26. ^{13}C NMR of additive XI

δ (ppm) (101 MHz, $CDCl_3$): 172.90 (C_9), 138.76 (C_6), 130.20 - 129.81 ($C_{16\text{-}17}$), from 128.43 to 126.30 ($C_{1\text{-}5}$), 40.30 (C_8), 36.65 (C_7), 35.54 (C_{10}), from 31.74 to 29.59 ($C_{12\text{-}14}$ $C_{19\text{-}25}$), 27.04 (C_{15} C_{18}), 22.50 (C_{11}), 13.93 (C_{26}).

4) Calculation of the Heithaus parameter for bitumen and for bitumen added with 25 wt% of additive (VII) by weight of bitumen.

The Heithaus parameter was obtained by titration method and is considered concluded when the flocculation point is reached. Once the volume of titrant is known, it is possible to calculate C, concentration of bitumen in the total volume, and FR, flocculation ratio, according to the relationships reported:

$$C = \frac{W_A}{V_S + V_T}$$
$$FR = \frac{V_S}{V_S + V_T}$$

Where W_A corresponds to the mass of bitumen in the sample, V_S to the volume of the solvent, toluene, and V_T to the volume of the titrant, *n*-heptane.
The C and FR data are plotted, the equation of the straight line is calculated by linear regression and C_{min} and FR_{max} are obtained as the intersection of the straight line respectively with the abscissa and ordinate axes.
For the bitumen alone (blank sample), data relating to the mass of bitumen in each sample, volume of solvent used, m/v % (g/mL) concentration of each sample, volume of titrant, C and FR are shown in Table S1 and Figure S29.

Table S1. Mass of bitumen, V_S, concentration, V_T, C and FR for three samples containing different concentrations of bitumen without additives.

**Sample**	**Bitumen (g)**	**Toluene (V_S) (mL)**	**Sample Conc. m/v% (g/mL)**	***n*-Heptane V_T (mL)**	**C (g/mL)**	**FR**
1	0.26	0.31	0.84	0.75	0.25	0.29
2	0.28	0.31	0.90	0.75	0.26	0.29
3	0.29	0.45	0.64	1.05	0.19	0.30
4	0.27	0.54	0.50	1.20	0.16	0.31

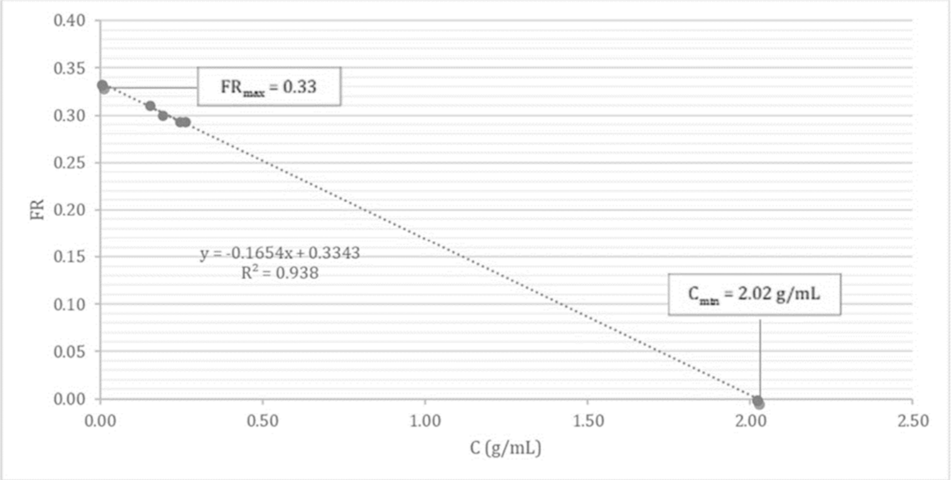

Figure S27. Straight line obtained by plotting the C values against the FR values for the bitumen samples without additives.

The linear correlation coefficient, R^2, is equal to 0.9380 and demonstrates the presence of a linear relationship between the different points. C_{min} equals 2.02 g/mL and FR_{max} equals 0.33. From these values of C_{min} and FR_{max}, P_a

was obtained, a parameter that describes the solubility of asphaltenes in bitumen, P_0, a parameter that defines the solvating power of maltenes in bitumen, and finally P, the Heithaus parameter.

$$P_a = 1 - FR_{max} = 1 - 0.33 = 0.67$$

$$P_0 = FR_{max} \cdot \left[\frac{1}{C_{min}} + 1\right] = 0.33 \cdot \left[\frac{1}{2.02\frac{g}{mL}} + 1\right] = 0.49$$

$$P = \frac{P_0}{1 - P_a} = \frac{0.49}{1 - 0.67} = 1.48$$

If P>1, then the bitumen is stable and does not tend to flocculate; if P<1, then the bitumen is unstable. Since P=1.48, it is concluded that, despite ageing and the absence of additives, the bitumen sample is stable
For bitumen containing 25 wt% of additive (**VII**) by weight of bitumen, data on the mass of bitumen in each sample, volume of solvent, m/v% concentration of each sample, volume of titrant, C and FR are shown in Table S2 and Figure S30.

Table S2. Mass of bitumen, V_S, concentration, V_T, C and FR for the four samples with different concentrations of bitumen containing 25 wt% of additive (**VII**).

**Sample**	**Bitumen (g)**	**Toluene V_S (mL)**	**Sample Conc. m/v% (g/mL)**	***n*-Heptane V_T (mL)**	**C (g/mL)**	**FR**
1	0.33	0.35	0.94	1.25	0.21	0.22
2	0.30	0.38	0.79	1.25	0.18	0.23
3	0.28	0.43	0.65	1.35	0.16	0.24
4	0.31	0.62	0.50	1.75	0.13	0.26

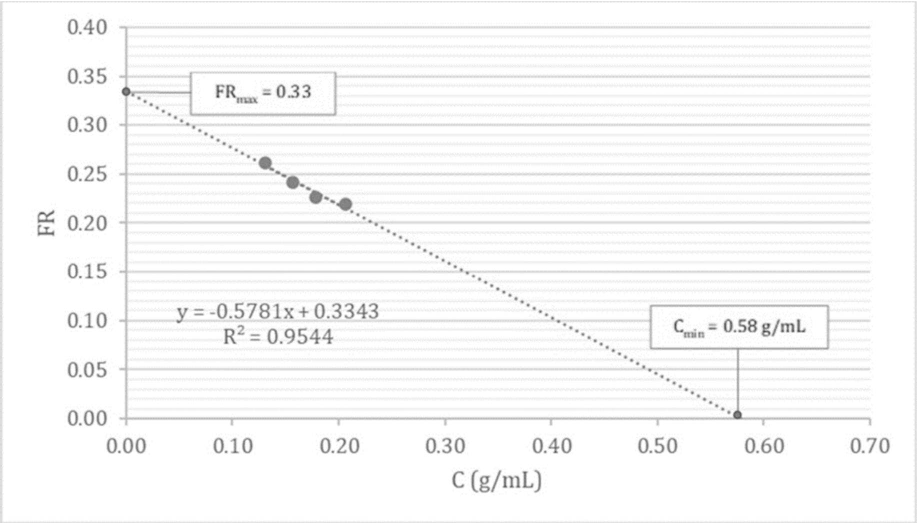

Figure S28. Straight line obtained by plotting the C values against the FR values for the bitumen samples containing 25 wt% of additive (**VII**).

The linear correlation coefficient. R^2. is equal to 0.9896 and demonstrates the presence of a linear relationship between the different points. C_{min} equals 0.58 g/mL and FR_{max} equals 0.33. By means of Equations above. P_a was obtained (0.67). a parameter that describes the solubility of asphaltenes in bitumen. P_0 (0.91). a parameter that defines the solvating power of maltenes in bitumen. and finally P. the Heithaus parameter. P = 2.73. Thus. additive bitumen does not tend to flocculate and is more stable than non-additive bitumen.
